# Supplementary figures and images for: Validating the performance of organ dysfunction scores in children with infection: A cohort study
Source: PLoS One. 2024 Jul 19;19(7):e0306172. doi: 10.1371/journal.pone.0306172 (PMC11259267; doi:10.1371/journal.pone.0306172)

variable

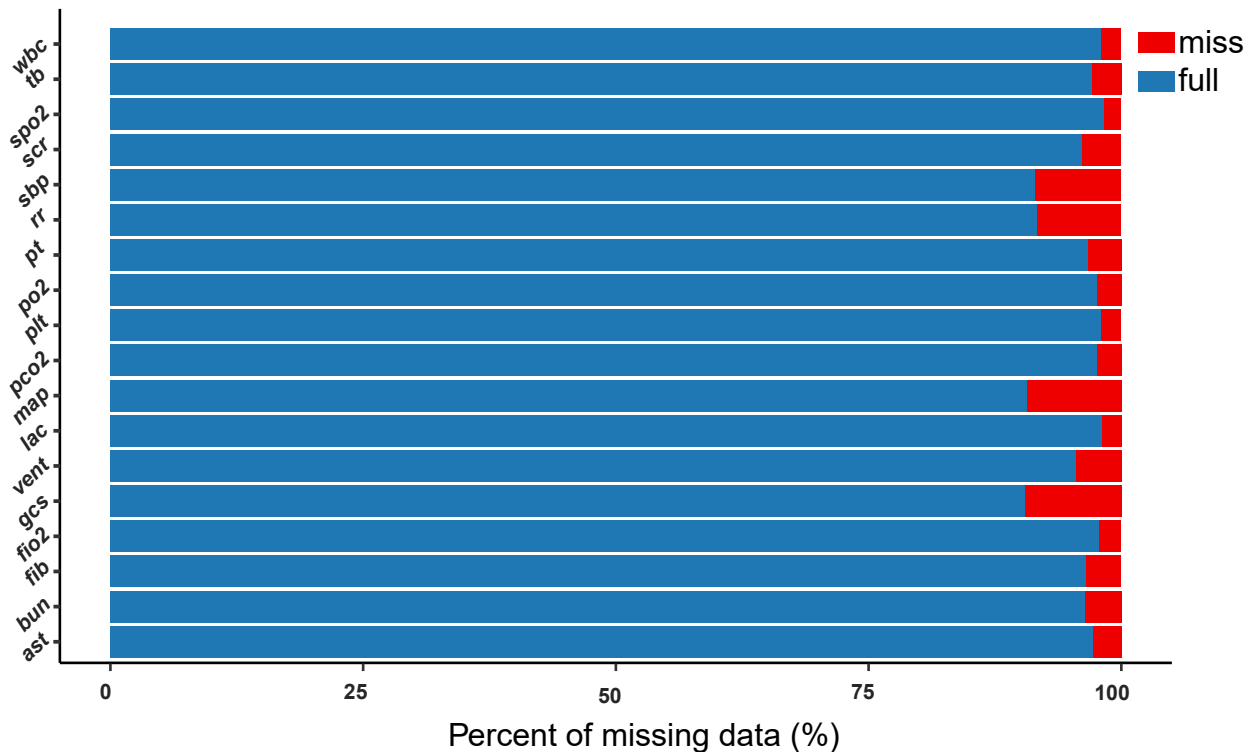

Supplement: S1 Fig — (PDF) [file pone.0306172.s001.pdf]

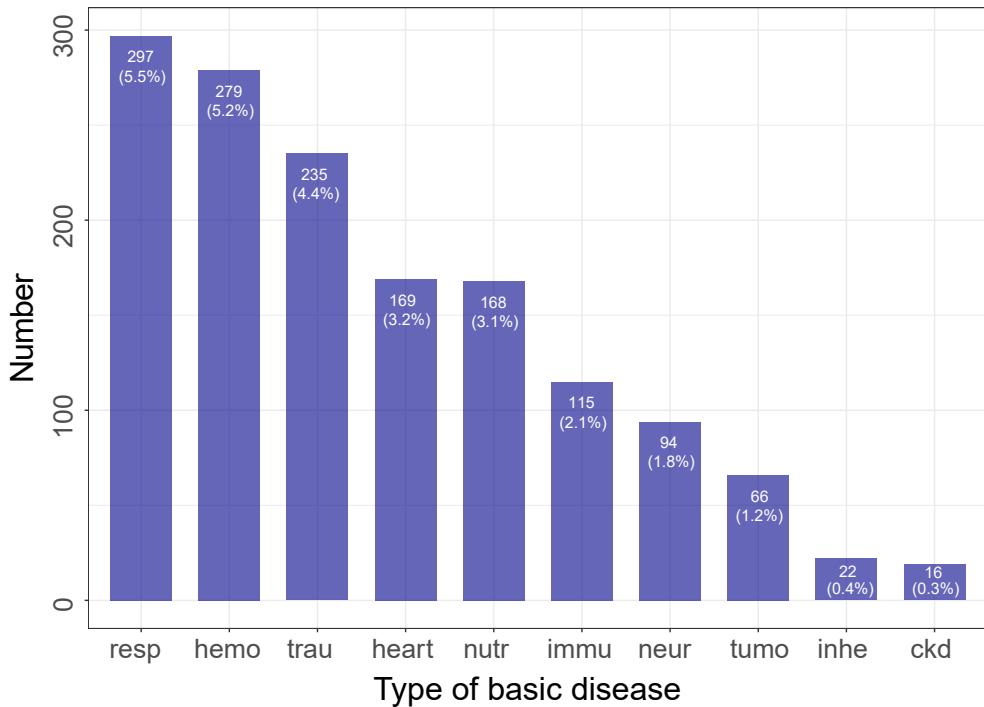

Supplement: S2 Fig — Note: The top three underlying diseases were respiratory diseases, hematological diseases and traumatic diseases. Abbreviations: resp, respiratory diseases; homo, hematological diseases; trau, traumatic diseases; heart, heart diseases; nutr, nutritional diseases; immu, autoimmune diseases; neur, neurological diseases; tumo, tumors; inhe, inherited metabolic disorders; CKD, chronic kidney disease. (PDF) [file pone.0306172.s002.pdf]

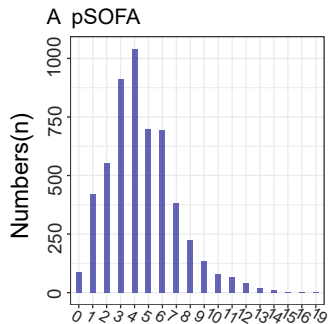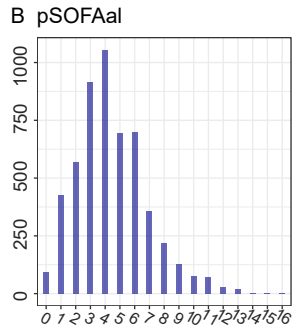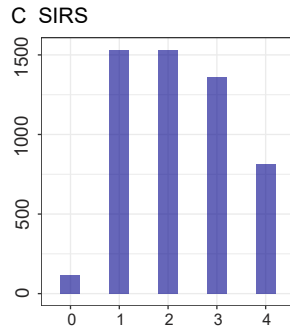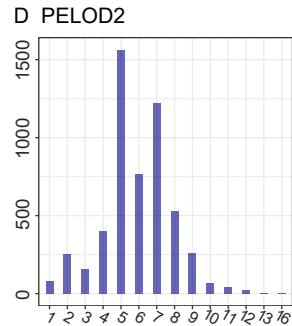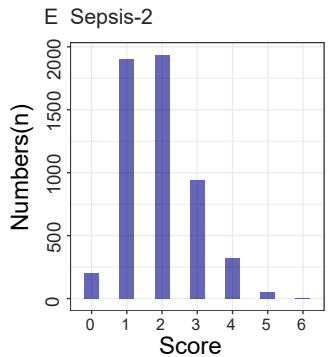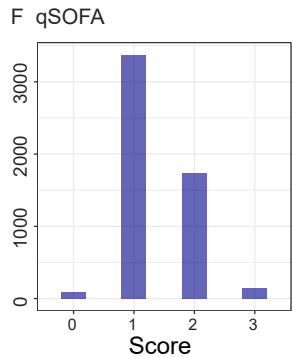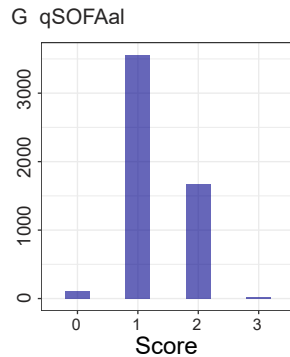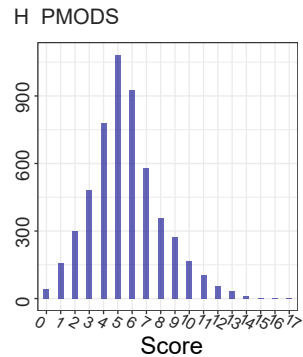

Supplement: S3 Fig — Note: The score distributions of the numbers of encounters are shown for A. pSOFA, B. pSOFAal, C. SIRS, D. PELOD2, E. Sepsis-2, F. qSOFA, G. qSOFAal, and H. PMODS. (PDF) [file pone.0306172.s003.pdf]

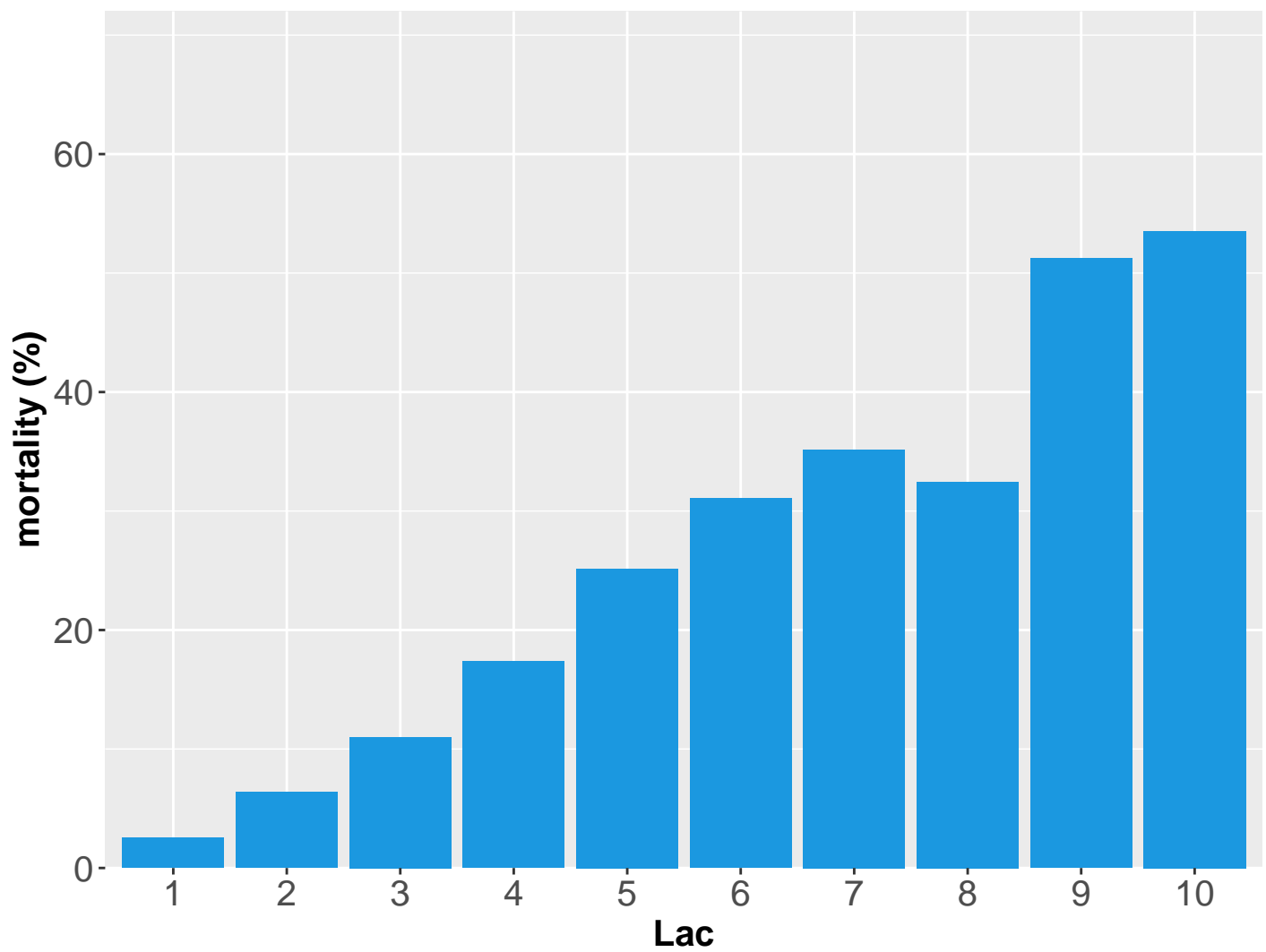

Supplement: S5 Fig — (PDF) [file pone.0306172.s005.pdf]

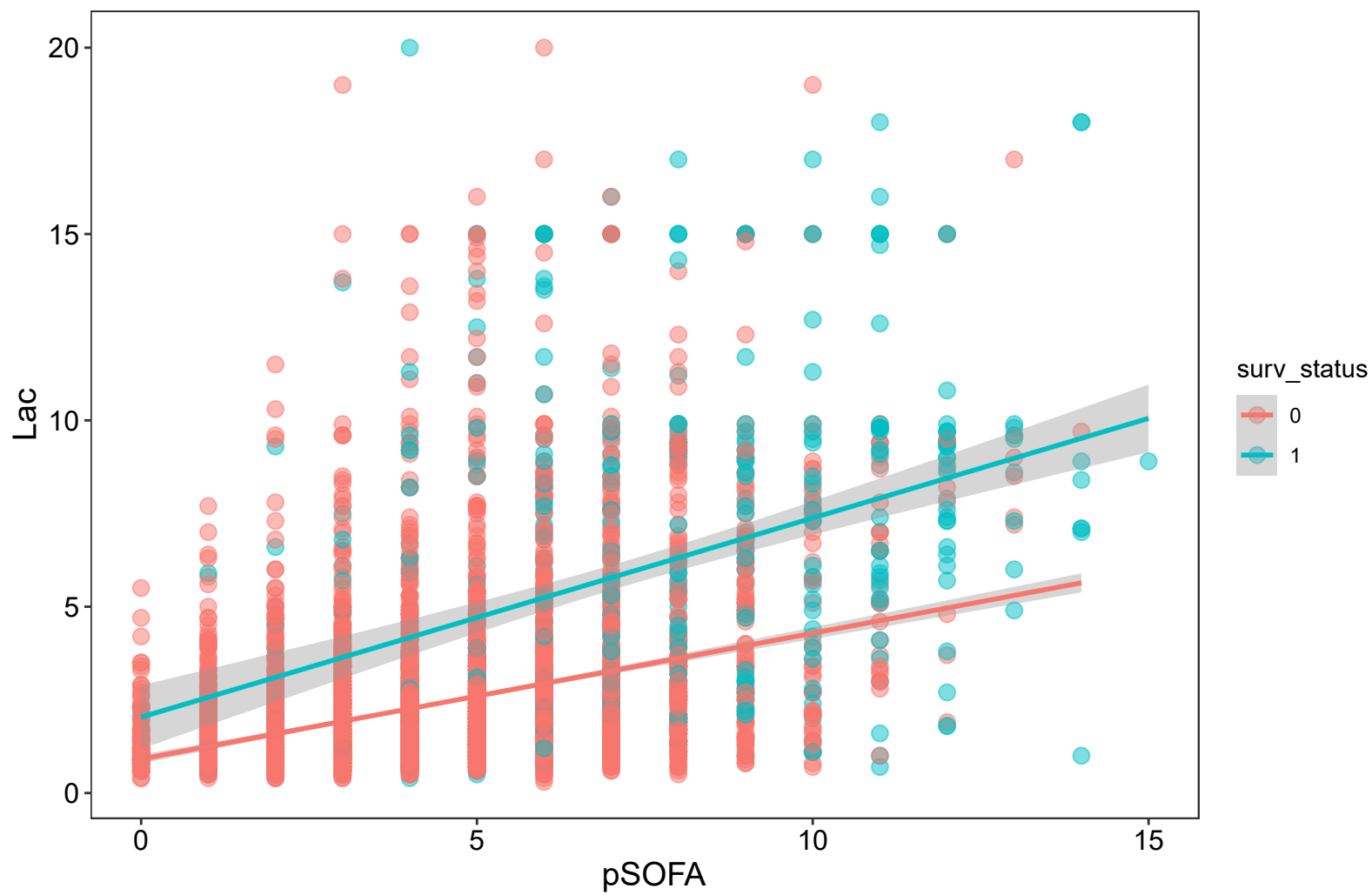

Supplement: S6 Fig — (PDF) [file pone.0306172.s006.pdf]

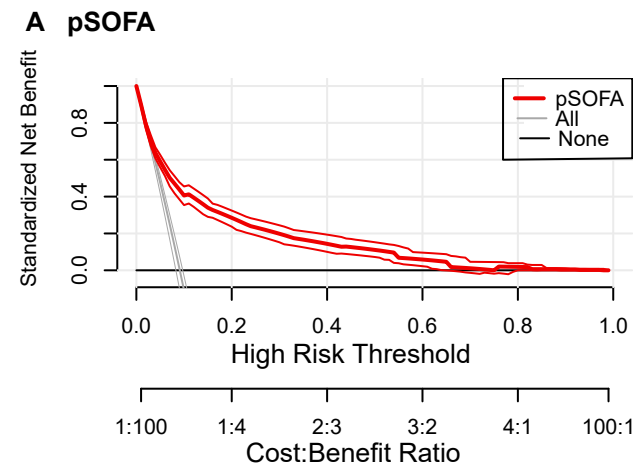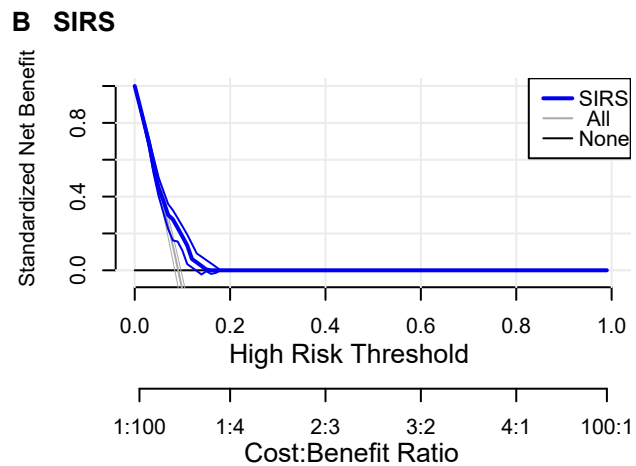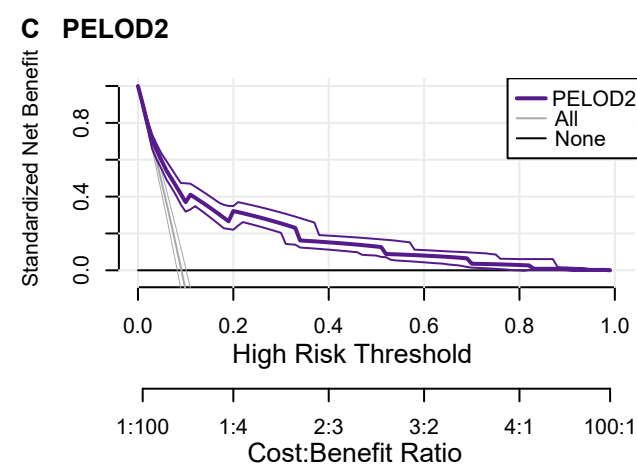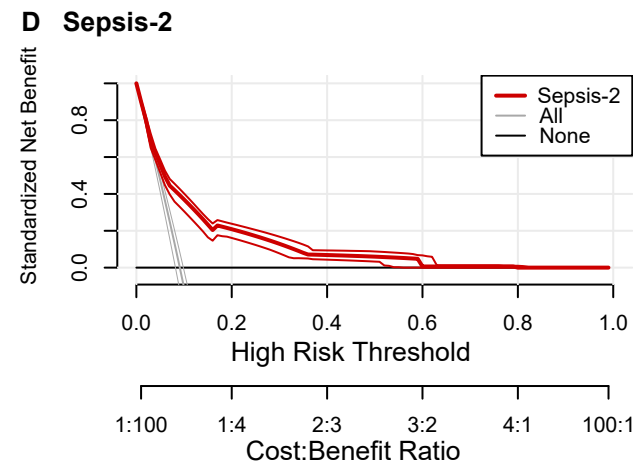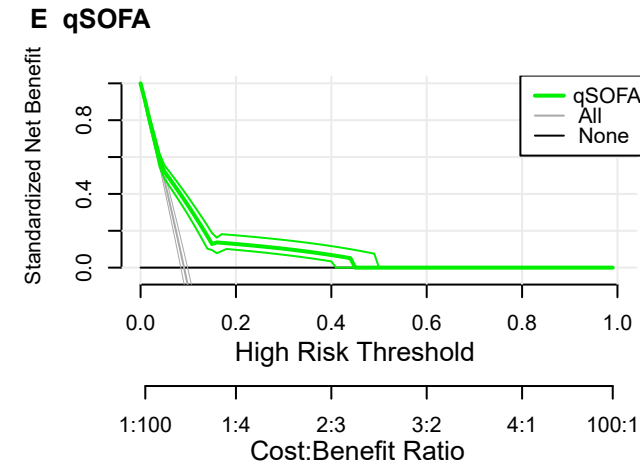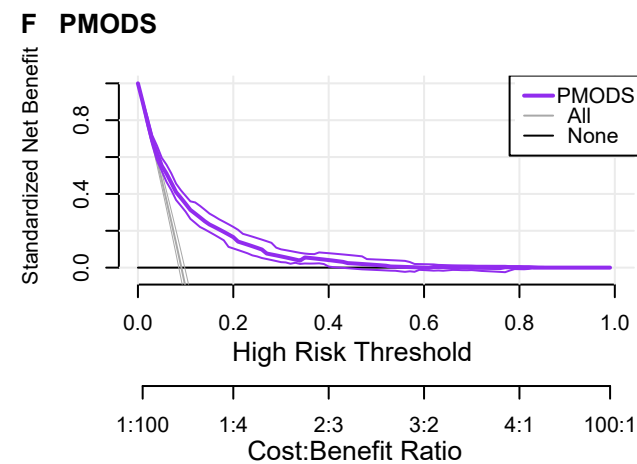

Supplement: S7 Fig — A. pSOFA, B. SIRS, C. PELOD2, D. Sepsis-2, E. qSOFA, F. PMODS. Note: DCAs show that the pSOFA and PELOD2 scores have superior net benefits compared to the other five scores. (PDF) [file pone.0306172.s007.pdf]

**A pSOFA**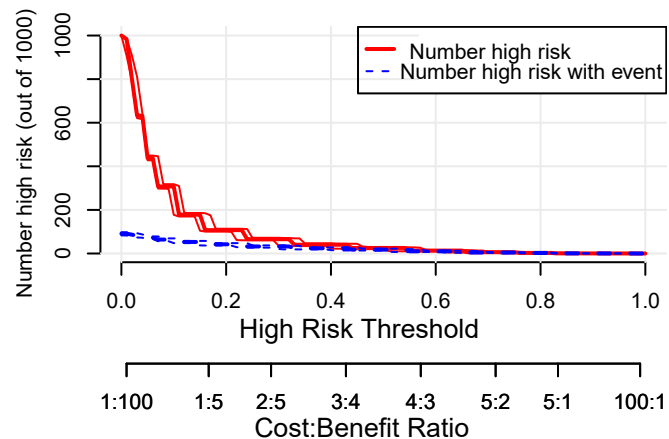**B SIRS**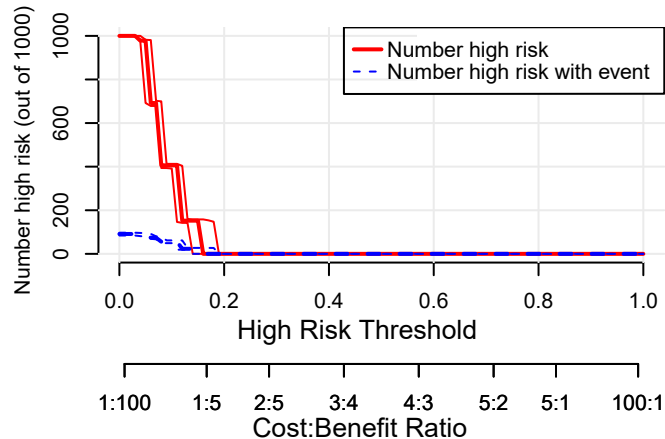**C PELOD2**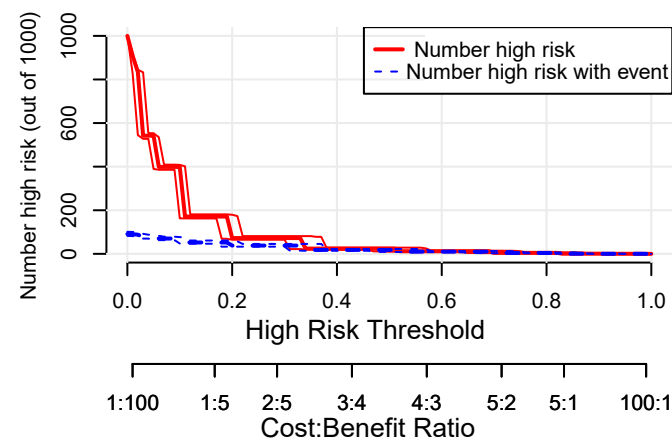**D Sepsis-2**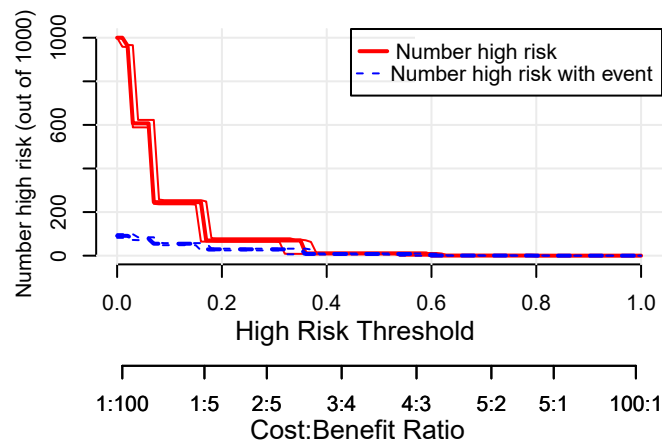**E qSOFA**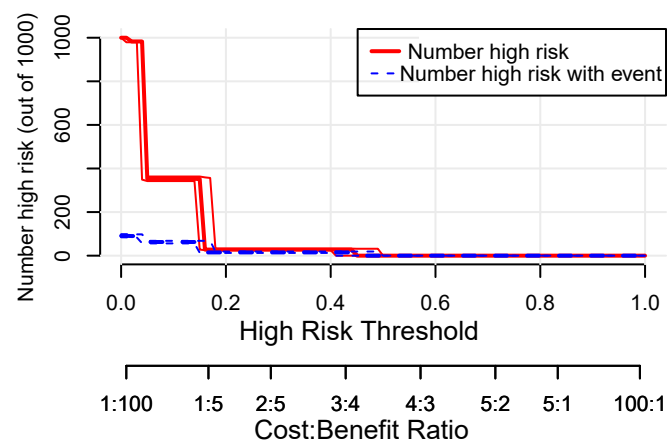**F PMODS**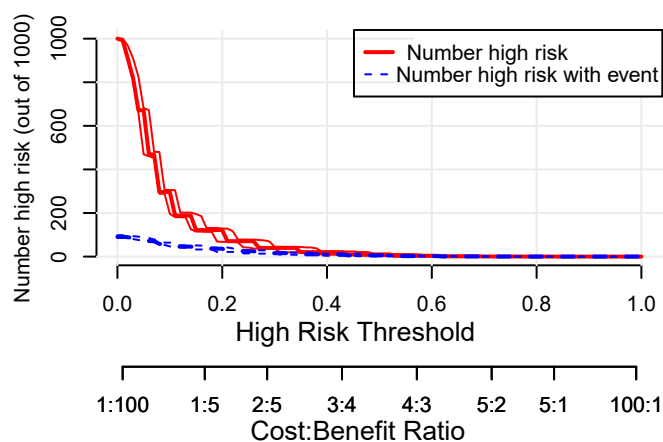

Supplement: S8 Fig — A. pSOFA, B. SIRS, C. PELOD2, D. Sepsis-2, E. qSOFA, F. PMODS. Note: The pSOFA and PELOD2 scores are clinically applicable for in-hospital mortality prediction. Abbreviations: CIC, clinical impact curve. (PDF) [file pone.0306172.s008.pdf]

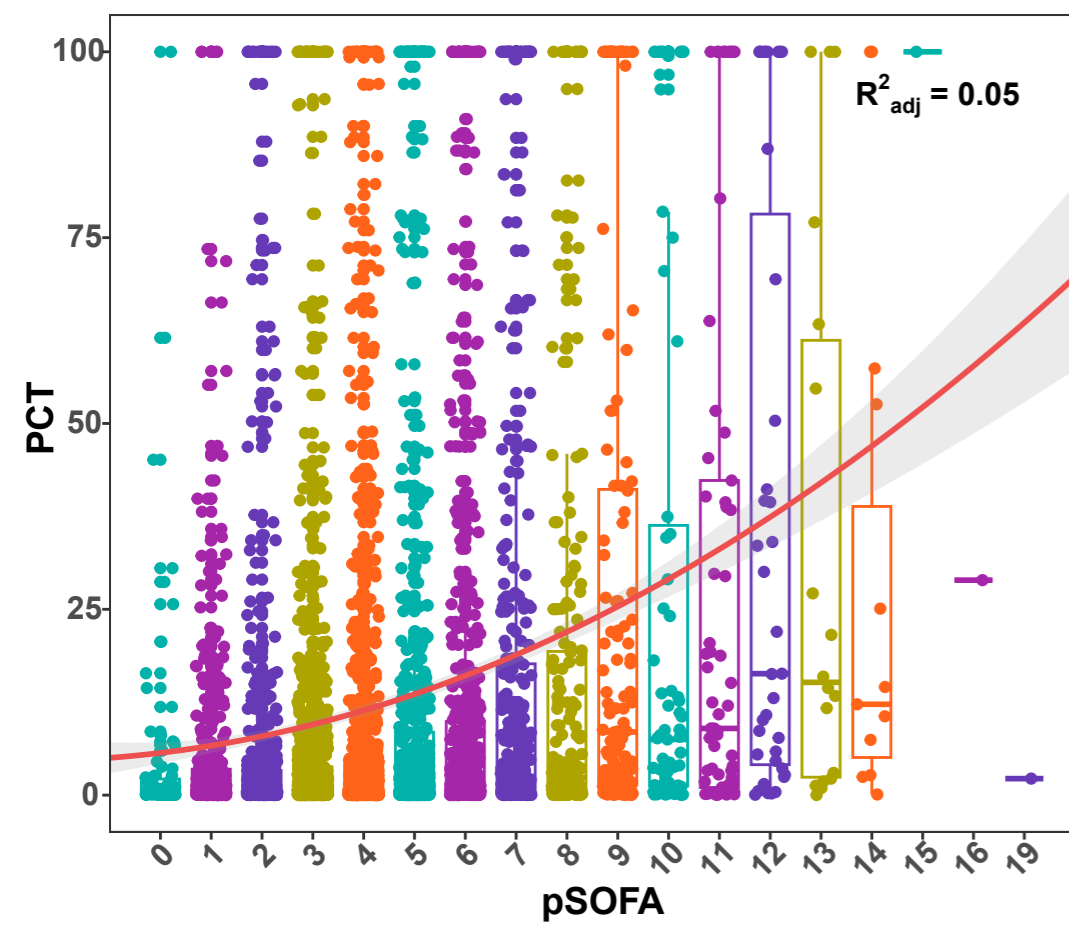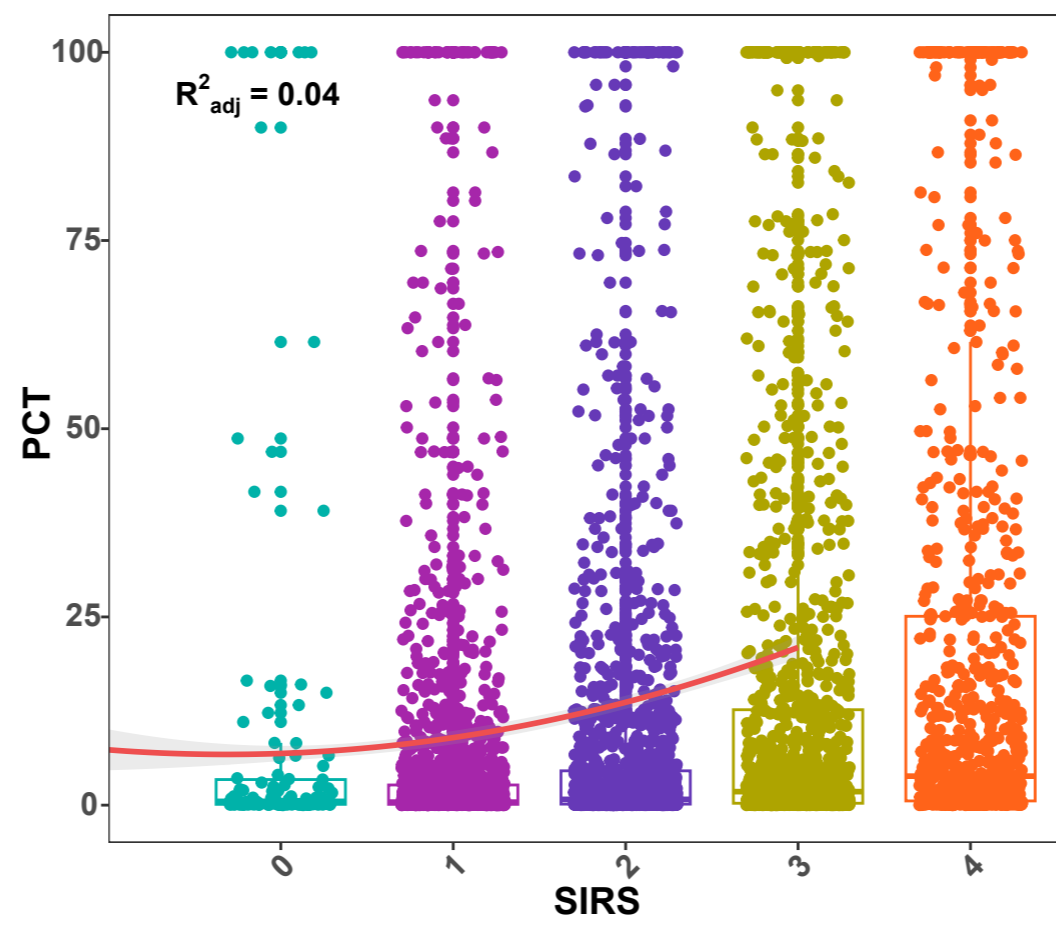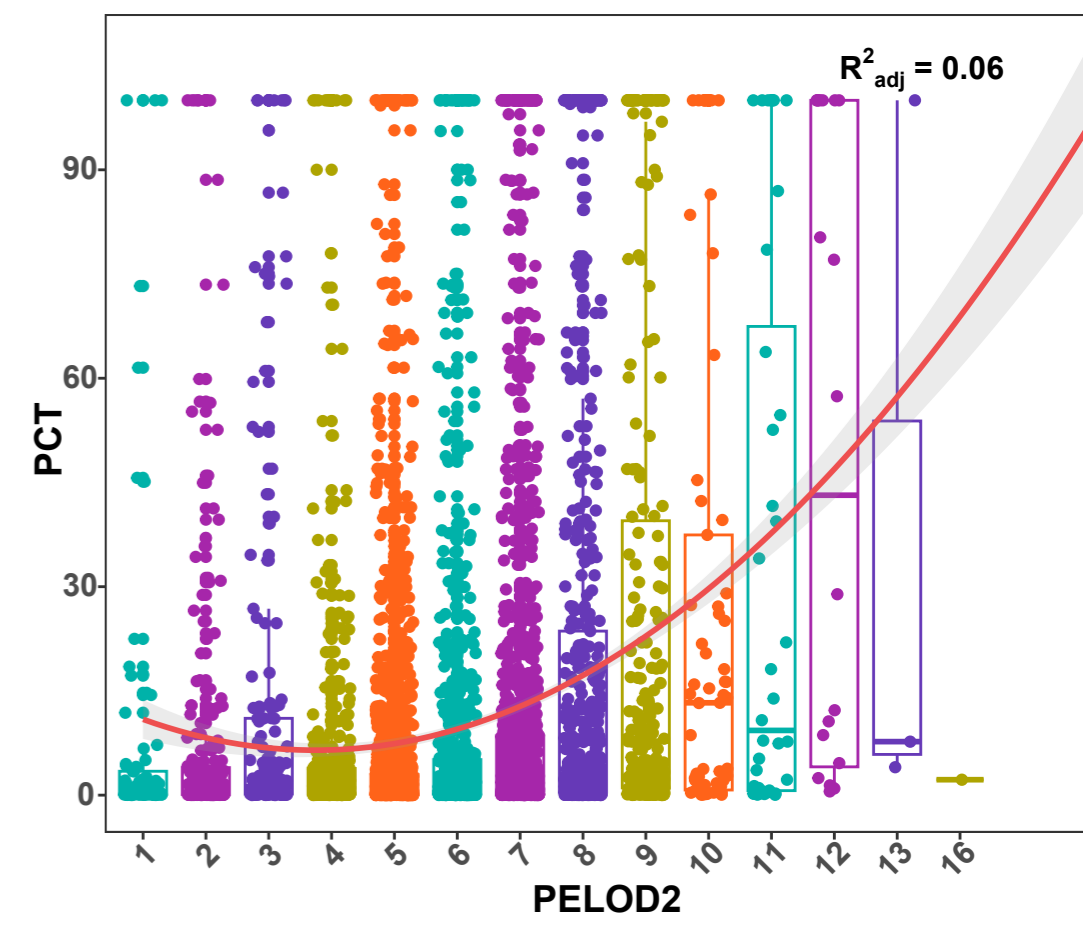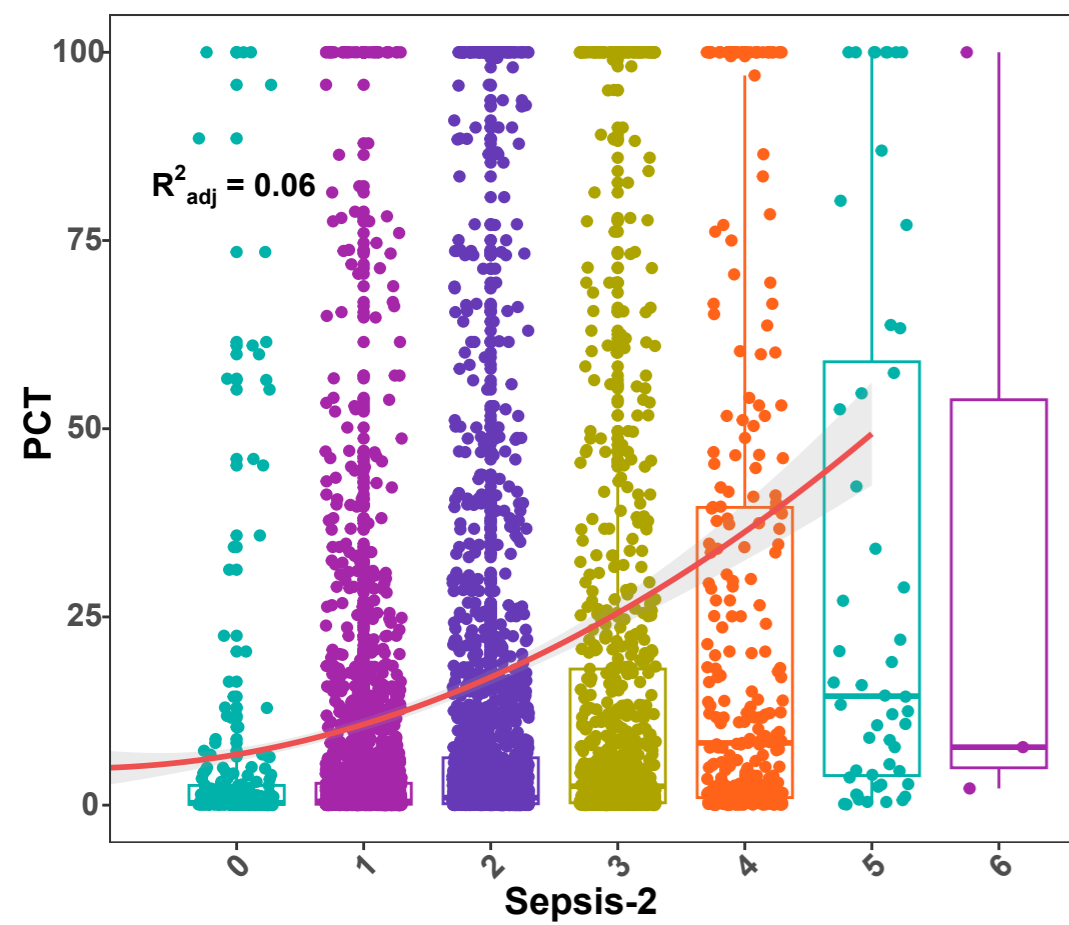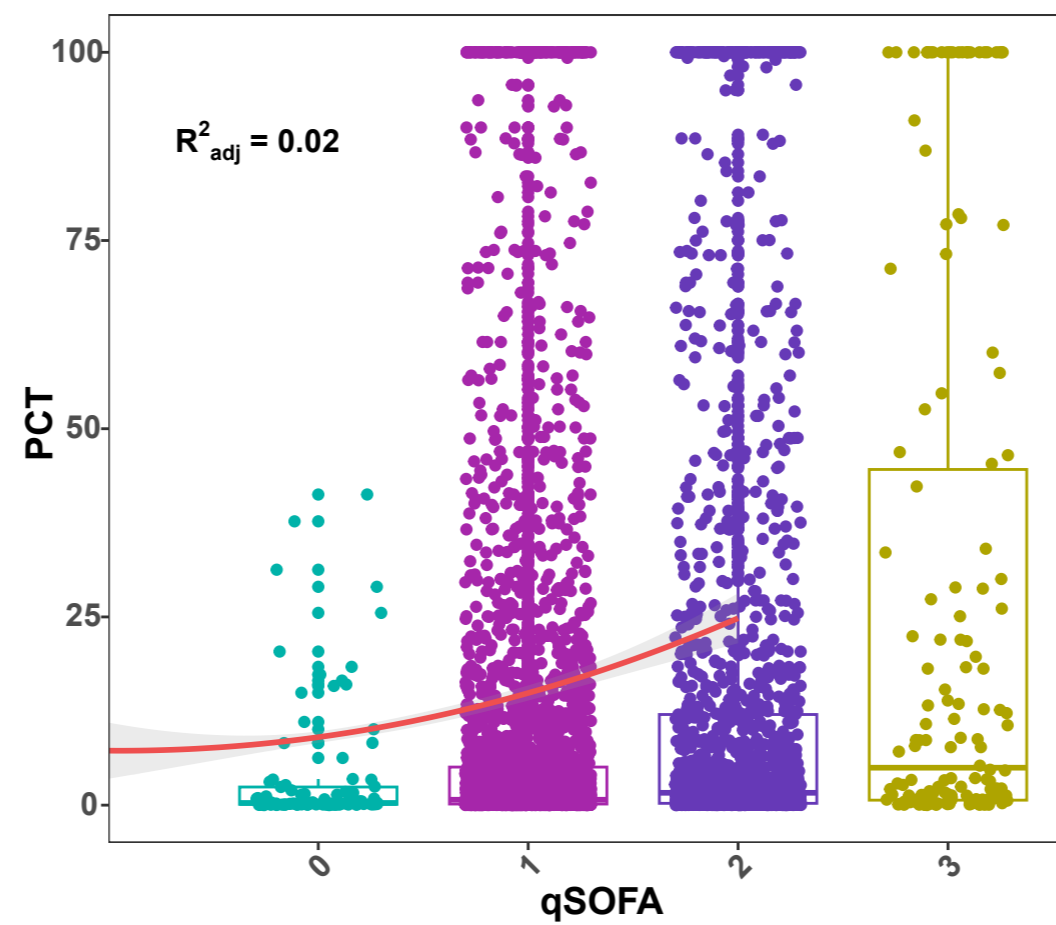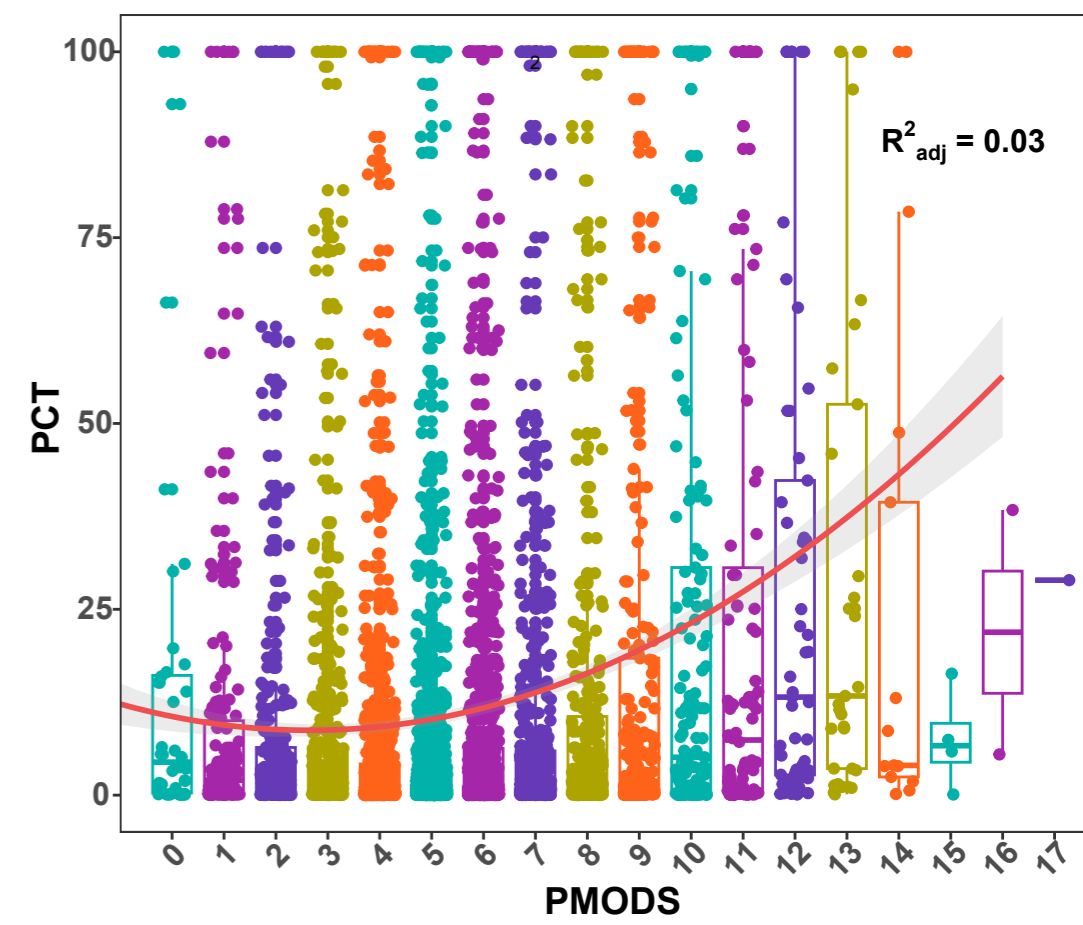

Supplement: S9 Fig — A. pSOFA vs. PCT, B. SIRS vs. PCT, C. PELOD2 vs. PCT, D. Sepsis-2 vs. PCT, E. qSOFA vs. PCT, F. PMODS vs. PCT. (PDF) [file pone.0306172.s009.pdf]

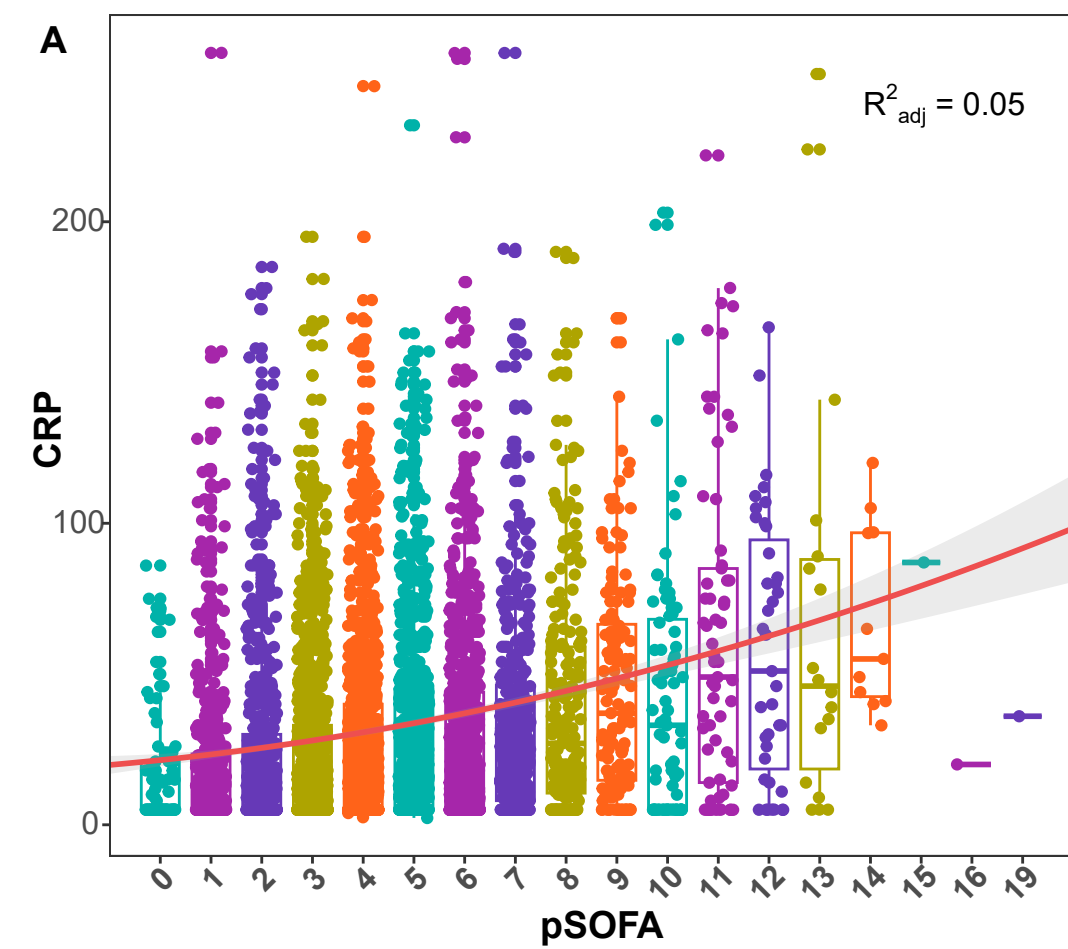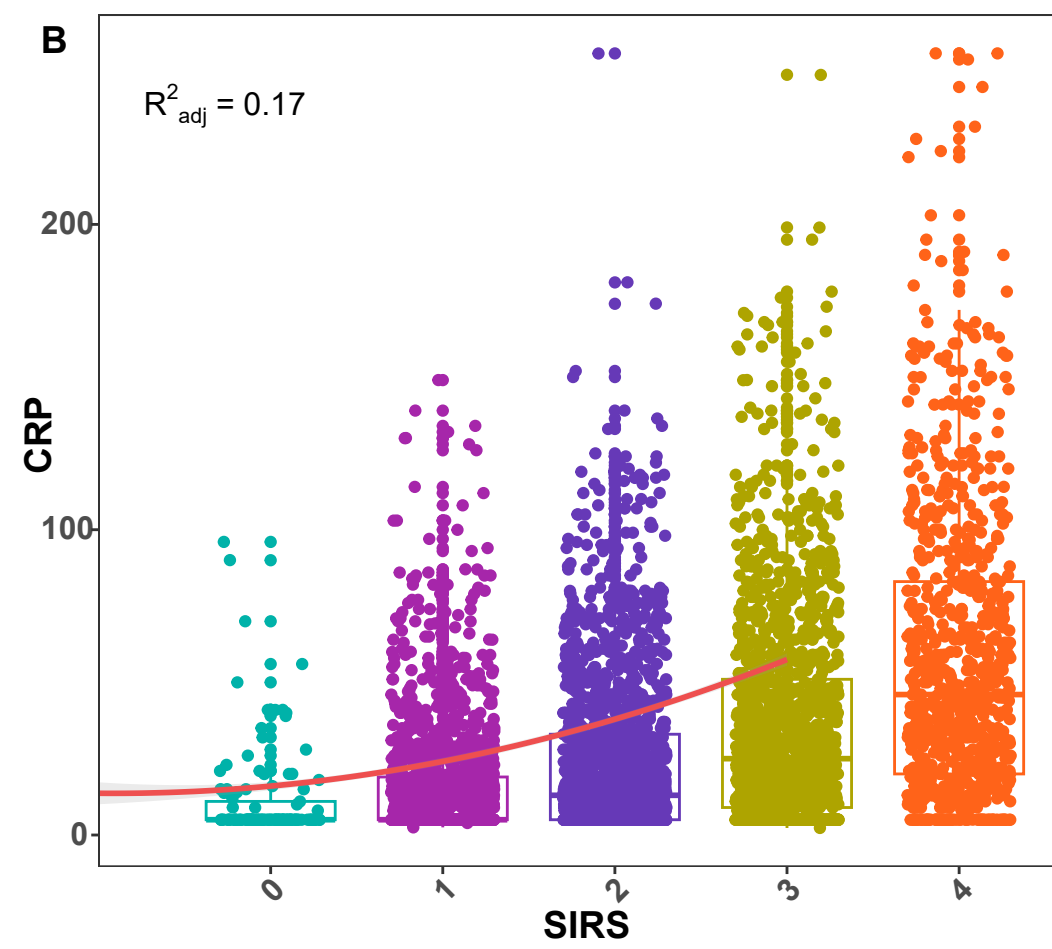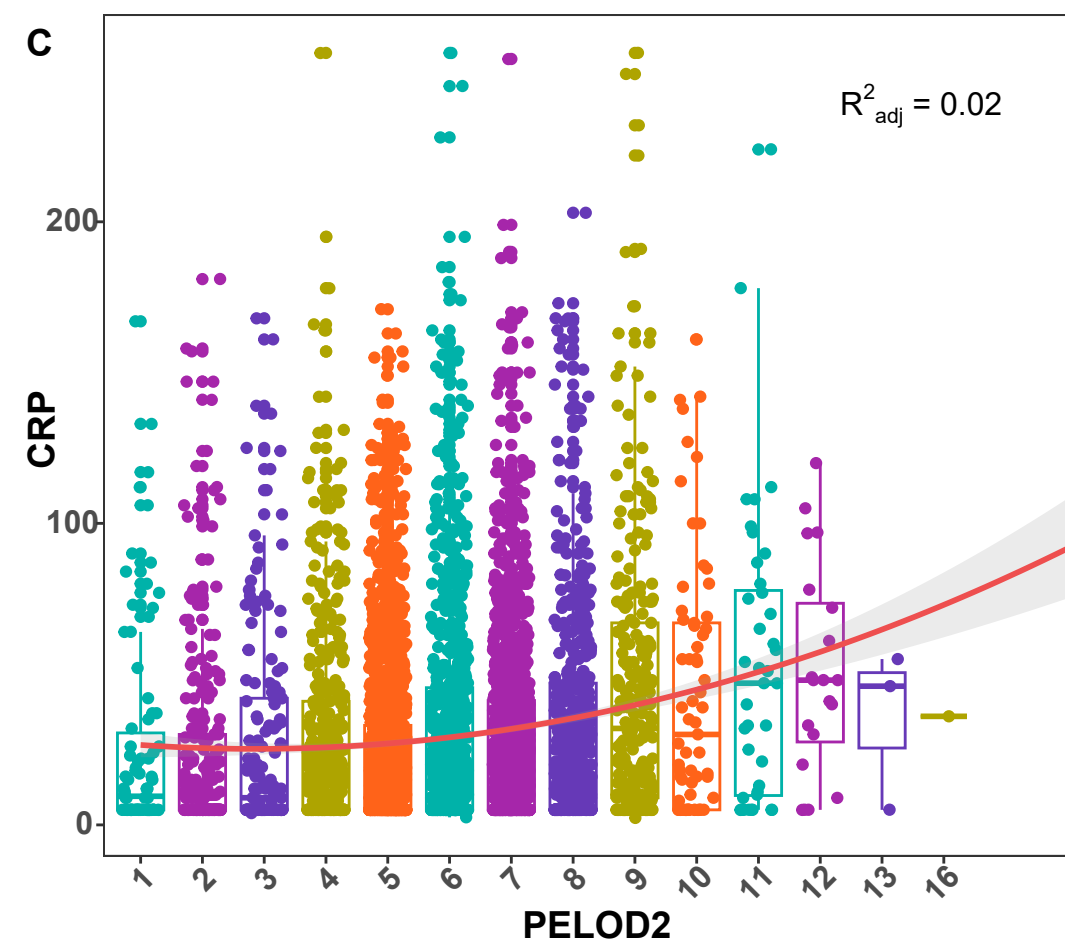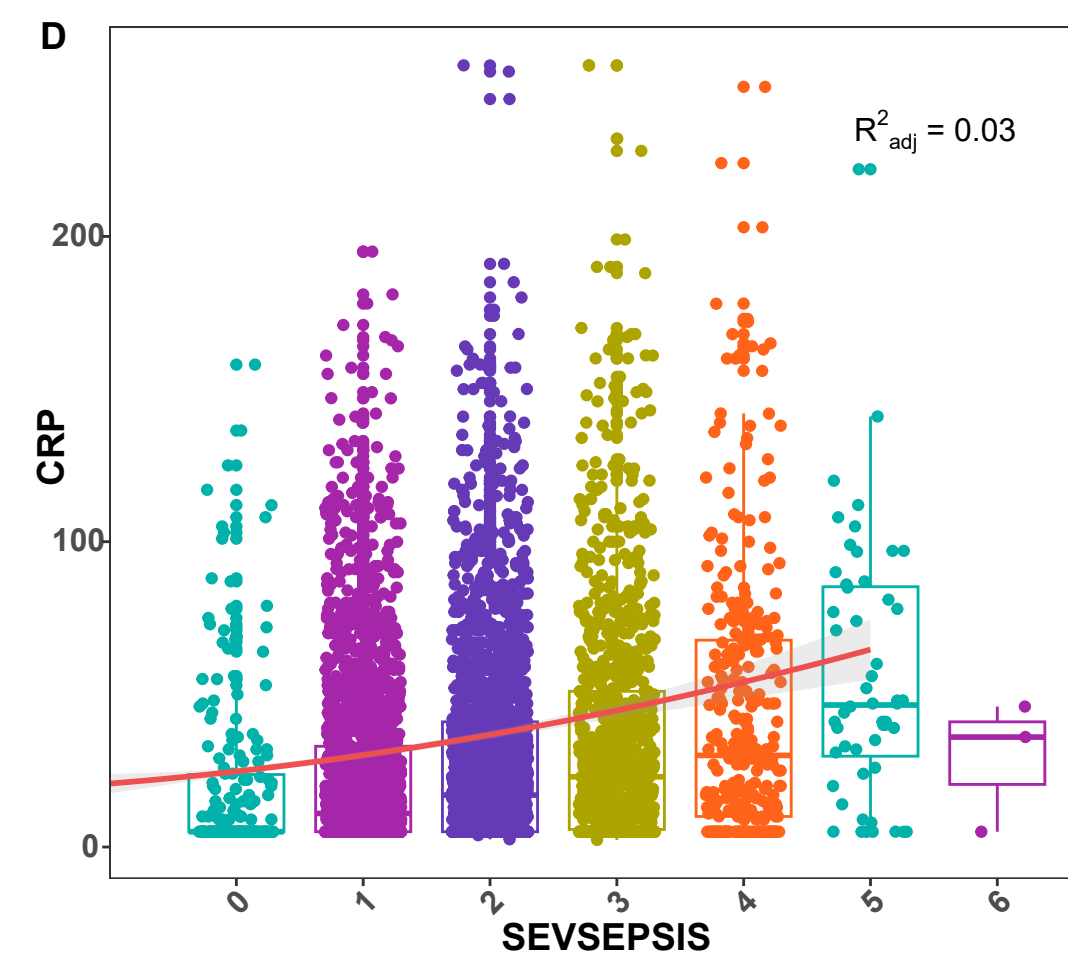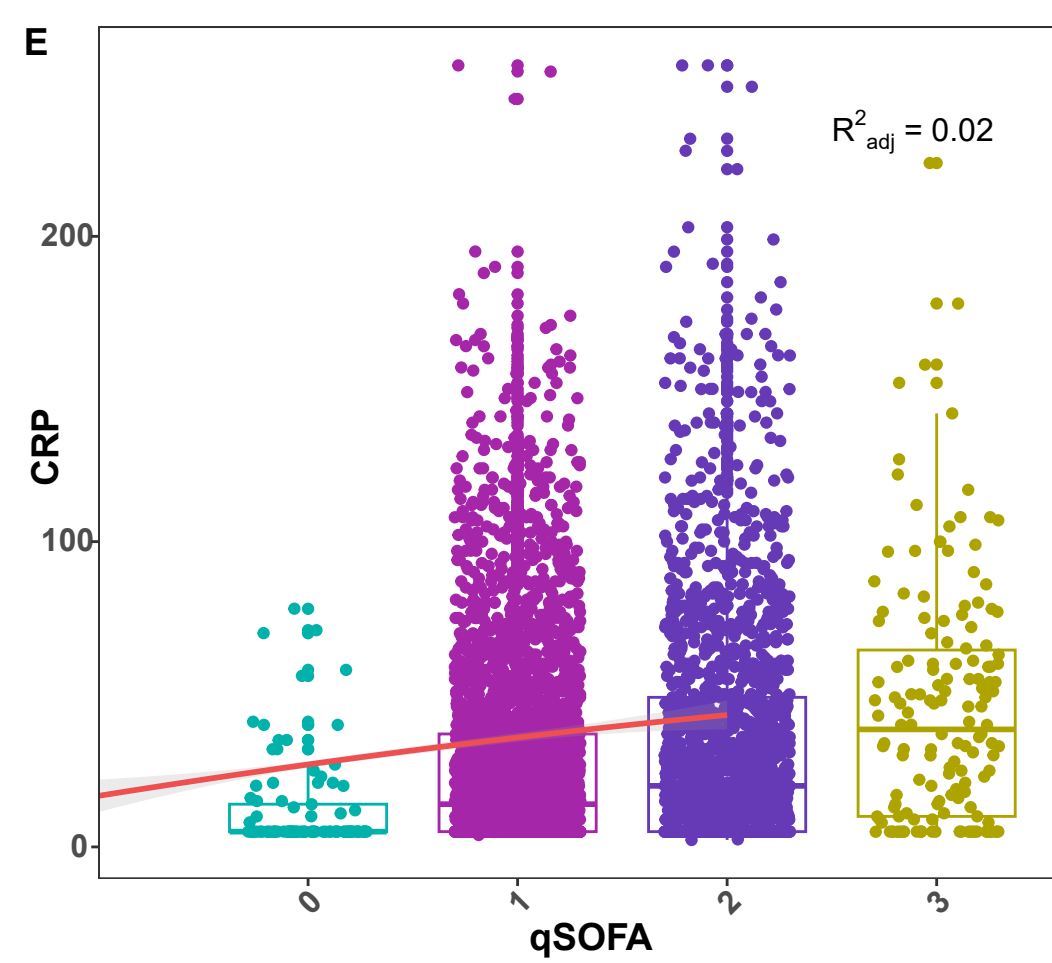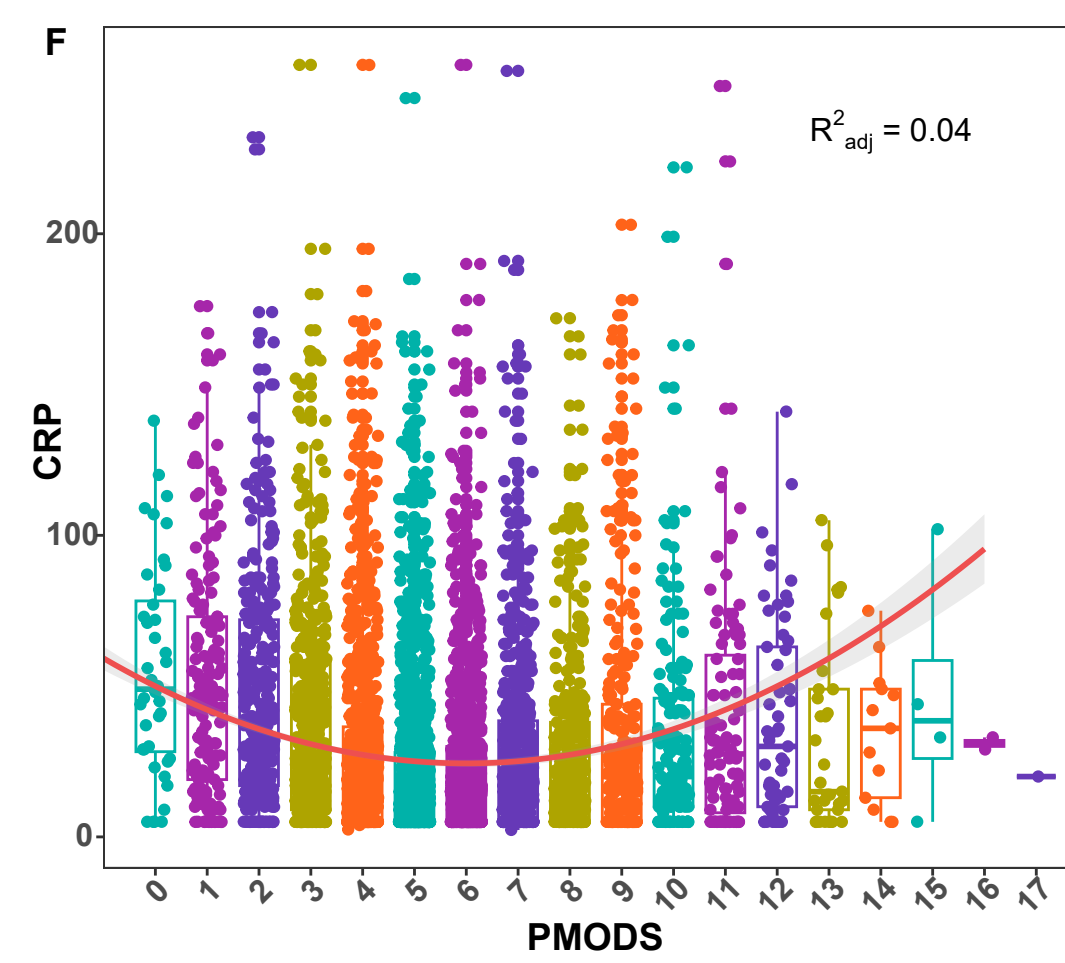

Supplement: S10 Fig — A. pSOFA vs. CRP, B. SIRS vs. CRP, C. PELOD2 vs. CRP, D. Sepsis-2 vs. CRP, E. qSOFA vs. CRP, F. PMODS vs. CRP. (PDF) [file pone.0306172.s010.pdf]
