# Supplementary material for: Validating the performance of organ dysfunction scores in children with infection: A cohort study
Source: PLoS One. 2024 Jul 19;19(7):e0306172. doi: 10.1371/journal.pone.0306172 (PMC11259267; doi:10.1371/journal.pone.0306172)
Supplement: S4 Fig — Note: Mortality is shown for A. the group aged less than 2 years, B. the group aged 2 years or older, C. the male group, D. the female group, E. the group with comorbidities, F. the group without comorbidities, and G. the total cohort. (PDF) [file pone.0306172.s004.pdf]

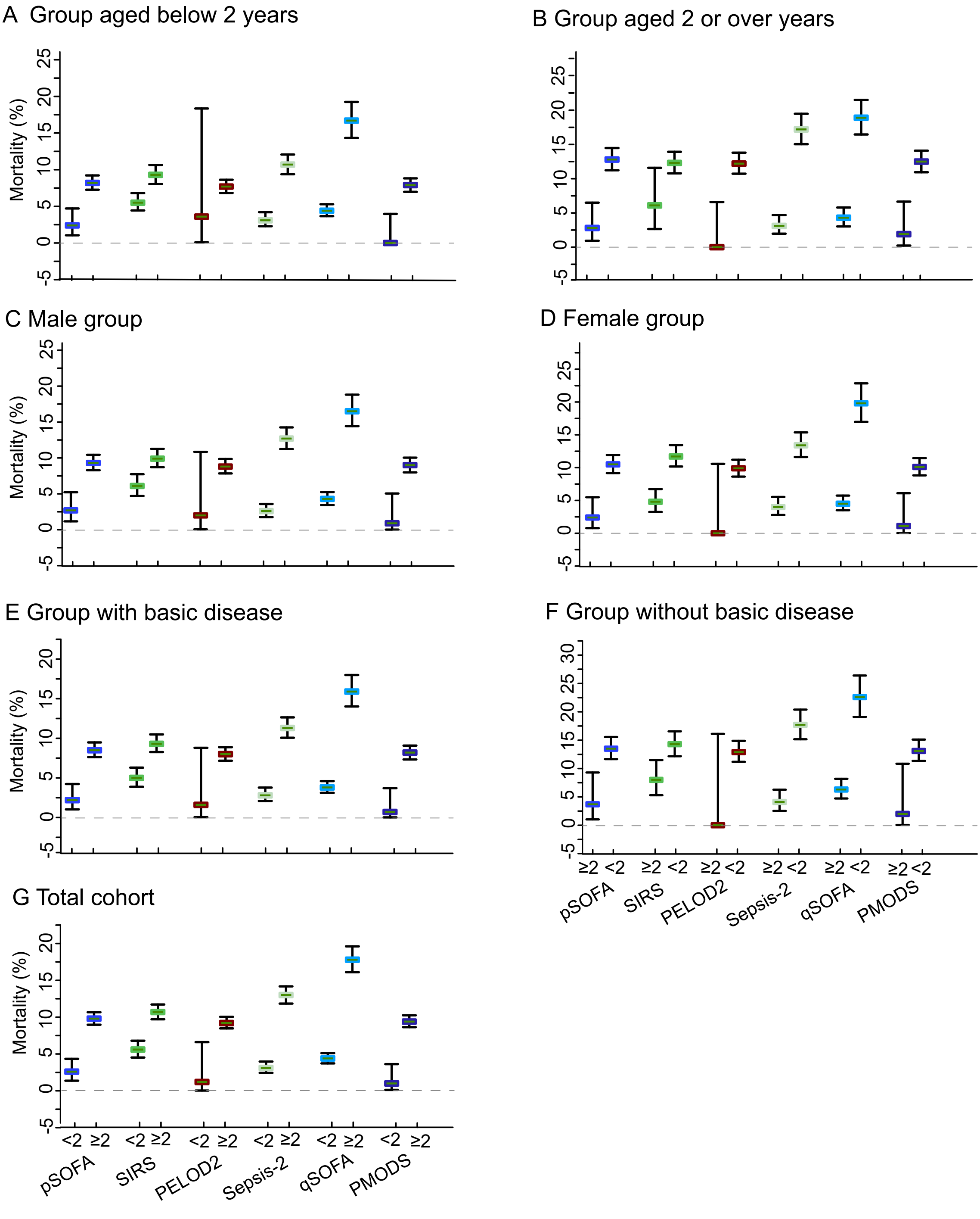
A Group aged below 2 years
B Group aged 2 or over years
C Male group
D Female group
E Group with basic disease
F Group without basic disease
G Total cohort
Mortality (%)
-5
0
5
10
15
20
25
30
<2 ≥2
≥2 <2
pSOFA
SIRS
PELOD2
Sepsis-2
qSOFA
PMODS
