# Supplementary material for: Validating the performance of organ dysfunction scores in children with infection: A cohort study
Source: PLoS One. 2024 Jul 19;19(7):e0306172. doi: 10.1371/journal.pone.0306172 (PMC11259267; doi:10.1371/journal.pone.0306172)
Supplement: S1 Table — (DOCX) [file pone.0306172.s011.docx]

**S1 Table. Scoring rules for the eight candidate models.**

**A. pSOFA**

| **Variables** | | **Score** | | | | |
| --- | --- | --- | --- | --- | --- | --- |
|  |  | **0** | **1** | **2** | **3** | **4** |
| **Respiratory** | |  |  |  |  |  |
|  | Pao2:Fio2 | ≥400 | <400 | <300 | <200 With respiratory support | <100 with respiratory support |
|  |  |  |  |  |  |  |
| **Coagulation** | |  |  |  |  |  |
|  | Platelet count (×10^9^/L) | ≥150 | 100-149 | 50-99 | 20-49 | <20 |
| **Hepatic** | |  |  |  |  |  |
|  | Bilirubin (mg/dL) | <1.2 | 1.2-1.9 | 2.0-5.9 | 6.0-11.9 | >12.0 |
| **Cardiovascular** | |  |  |  |  |  |
|  | MAP (mmHg) |  |  |  |  |  |
|  | <2 years | ≥60 | 44-59 | 31-43 |  | ≤30 |
|  | 2 to 5 yrs | ≥62 | 46-61 | 32-44 |  | ≤31 |
|  | >5 to 12 yrs | ≥65 | 49-64 | 36-48 |  | ≤35 |
|  | >12 to <18 yrs | ≥67 | 52-66 | 38-51 |  | ≤37 |
| **Neurologic** | |  |  |  |  |  |
|  | Glasgow Coma Score | 15 | 13-14 | 10-12 | 6-9 | <6 |
| **Renal** | |  |  |  |  |  |
|  | Creatinine (mg/dL) |  |  |  |  |  |
|  | <2 years | ≤34 |  | ≥35 |  |  |
|  | 2 to 5 yrs | ≤50 |  | ≥51 |  |  |
|  | >5 to 12 yrs | ≤58 |  | ≥59 |  |  |
|  | >12 to <18 yrs | ≤92 |  | ≥93 |  |  |

**B. Alternate pSOFA(pSOFAal)**

| **Variables** | | **Score** | | | | |
| --- | --- | --- | --- | --- | --- | --- |
|  |  | **0** | **1** | **2** | **3** | **4** |
| **Respiratory** | |  |  |  |  |  |
|  | Pao2:Fio2 | ≥400 | <400 | <300 | <200 With respiratory support | <100 with respiratory support |
|  |  |  |  |  |  |  |
| **Coagulation** | |  |  |  |  |  |
|  | Platelet count (×10^9^/L) | ≥150 | 100-149 | 50-99 | 20-49 | <20 |
| **Hepatic** | |  |  |  |  |  |
|  | Bilirubin (mg/dL) | <1.2 | 1.2-1.9 | 2.0-5.9 | 6.0-11.9 | >12.0 |
| **Cardiovascular** | |  |  |  |  |  |
|  | SBP (mmHg) |  |  |  |  |  |
|  | <2 years | ≥75 | <75 |  |  |  |
|  | 2 to 5 yrs | ≥74 | <74 |  |  |  |
|  | >5 to 12 yrs | ≥83 | <83 |  |  |  |
|  | >12 to <18 yrs | ≥90 | <90 |  |  |  |
| **Neurologic** | |  |  |  |  |  |
|  | Glasgow Coma Score | 15 | 13-14 | 10-12 | 6-9 | <6 |
| **Renal** | |  |  |  |  |  |
|  | Creatinine (mg/dL) |  |  |  |  |  |
|  | <2 years | ≤34 |  | ≥35 |  |  |
|  | 2 to 5 yrs | ≤50 |  | ≥51 |  |  |
|  | >5 to 12 yrs | ≤58 |  | ≥59 |  |  |
|  | >12 to <18 yrs | ≤92 |  | ≥93 |  |  |

**C. SIRS**

| **Variables** | | **Score** | |
| --- | --- | --- | --- |
|  |  | 0 | 1 |
| **<2 years** | |  |  |
|  | Heart Rate | 90-180 | <90 or >180 |
|  | Respiratory Rate | ≤34 | >34 |
|  | Leukocyte Count (×10^9^/L) | 5-17.5 | >17.5 or >5 |
|  | Temperature (℃) | 36-38.5 | <36 or >38.5 |
| **2 to 5 years** | |  |  |
|  | Heart Rate | ≤140 | >140 |
|  | Respiratory Rate | ≤22 | >22 |
|  | Leukocyte Count (×10^9^/L) | 6-15.5 | >15.5 or <6 |
|  | Temperature (℃) | 36-38.5 | <36 or >38.5 |
| **>5 to 12 years** | |  |  |
|  | Heart Rate | ≤130 | >130 |
|  | Respiratory Rate | ≤18 | >18 |
|  | Leukocyte Count (×10^9^/L) | 4.5-13.5 | >13.5 or <4.5 |
|  | Temperature (℃) | 36-38.5 | <36 or >38.5 |
| **>12 to <18 years** | |  |  |
|  | Heart Rate | ≤110 | >110 |
|  | Respiratory Rate | ≤14 | >14 |
|  | Leukocyte Count (×10^9^/L) | 4.5-11 | >11 or <4.5 |
|  | Temperature (℃) | 36-38.5 | <36 or >38.5 |

**D. PELOD 2**

| **Variables** | | **Score** | | | | | | |
| --- | --- | --- | --- | --- | --- | --- | --- | --- |
|  |  | **0** | **1** | **2** | **3** | **4** | **5** | **6** |
| **Central nervous system** | |  |  |  |  |  |  |  |
|  | Glasgow coma scale | 15 | 13-14 | 10-12 | 6-9 | <6 |  |  |
| **Cardiovascular** | |  |  |  |  |  |  |  |
|  | Lactatemia(mmol/L) | <5.0 | 5.0-10.7 |  |  | ≥11 |  |  |
|  | MAP (mmHg) |  |  |  |  |  |  |  |
|  | <2 years | ≥60 |  | 44-59 | 31-43 |  |  | ≤30 |
|  | 2 to 5 yrs | ≥62 |  | 46-61 | 32-44 |  |  | ≤31 |
|  | >5 to 12 yrs | ≥65 |  | 49-64 | 36-48 |  |  | ≤35 |
|  | >12 to <18 yrs | ≥67 |  | 52-66 | 38-51 |  |  | ≤37 |
| **Renal** | |  |  |  |  |  |  |  |
|  | Creatinine (mg/dL) |  |  |  |  |  |  |  |
|  | <2 years | ≤34 |  | ≥35 |  |  |  |  |
|  | 2 to 5 yrs | ≤50 |  | ≥51 |  |  |  |  |
|  | >5 to 12 yrs | ≤58 |  | ≥59 |  |  |  |  |
|  | >12 to <18 yrs | ≤92 |  | ≥93 |  |  |  |  |
| **Respiration** | |  |  |  |  |  |  |  |
|  | PaO2/FiO2 | ≥61 |  | <60 |  |  |  |  |
|  | PaCO2 (mmHg) | ≤58 | 59-94 |  | ≥95 |  |  |  |
|  | Invasive ventilation | No |  |  | Yes |  |  |  |
| **Hematologic** | |  |  |  |  |  |  |  |
|  | White cell count (×10^9^/L) | >2 |  | ≤2 |  |  |  |  |
|  | Platelets (×10^9^/L) | >142 | 77-141 | ≤76 |  |  |  |  |

**E. Sepsis-2**

| **Variables** | | **Score** | |
| --- | --- | --- | --- |
|  |  | 0 | 1 |
| **Respiration** | |  |  |
|  | PaO2/FiO2 (mmHg) | ≥300 | <300 |
|  | PaCO2 (mmHg) | ≤65 | >65 |
|  | Invasive ventilation | No | Yes |
|  | Non-Invasive ventilation | No | Yes |
| **Cardiovascular** | |  |  |
|  | SBP(mmHg) | ≥75 | <75 |
|  | <2 years | ≥74 | <74 |
|  | 2 to 5 yrs | ≥83 | <83 |
|  | >5 to 12 yrs | ≥90 | <90 |
|  | >12 to <18 yrs |  |  |
| **Renal** | |  |  |
|  | Creatinine (mg/dL) |  |  |
|  | <2 years | ≤34 | >34 |
|  | 2 to 5 yrs | ≤50 | >50 |
|  | >5 to 12 yrs | ≤58 | >58 |
|  | >12 to <18 yrs | ≤92 | >92 |
| **Hepatic** | |  |  |
|  | Bilirubin (umol/l) | <20 | ≥20 |
| **Haematologic** | |  |  |
|  | Platelets (x10(9)/L) | ≥80 | <80 |
| **Central nervous system** | |  |  |
|  | Glasgow coma scale | ≥12 | <12 |

**F. qSOFA**

| **Variables** | | **Score** | |
| --- | --- | --- | --- |
|  |  | 0 | 1 |
| **Tachypnea** | |  |  |
|  | Respiratory Rate |  |  |
|  | <2 years | ≥34 | >34 |
|  | 2 to 5 yrs | ≥22 | >22 |
|  | >5 to 12 yrs | ≥18 | >18 |
|  | >12 to <18 yrs | ≥14 | >14 |
| **Altered mentation** | |  |  |
|  | Glasgow coma scale | 15 | <15 |
| **Arterial hypotension** | |  |  |
|  | MAP (mmHg) |  |  |
|  | <2 years | ≥60 | <60 |
|  | 2 to 5 yrs | ≥62 | <62 |
|  | >5 to 12 yrs | ≥65 | <65 |
|  | >12 to <18 yrs | ≥67 | <67 |

**G. Alternate qSOFA(qSOFAal)**

| **Variables** | | **Score** | |
| --- | --- | --- | --- |
|  |  | 0 | 1 |
| **Tachypnea** | |  |  |
|  | Respiratory Rate |  |  |
|  | <2 years | ≥34 | >34 |
|  | 2 to 5 yrs | ≥22 | >22 |
|  | >5 to 12 yrs | ≥18 | >18 |
|  | >12 to <18 yrs | ≥14 | >14 |
| **Altered mentation** | |  |  |
|  | Glasgow coma scale | 15 | <15 |
| **Arterial hypotension** | |  |  |
|  | SBP (mmHg) |  |  |
|  | <2 years | ≥75 | <75 |
|  | 2 to 5 yrs | ≥74 | <74 |
|  | >5 to 12 yrs | ≥83 | <83 |
|  | >12 to <18 yrs | ≥90 | <90 |

**H. PMODS**

| **Variables** | **Score** | | | | |
| --- | --- | --- | --- | --- | --- |
|  | **0** | **1** | **2** | **3** | **4** |
| Lactic acid (mmol/L) | <1 | 1-2 | 2-5 | 5-7.5 | >7.5 |
| PaO2/FIO2 | >150 | 150-100 | 100-75 | 75-50 | <50 |
| Bilirubin (mg/dL) | <0.5 | 0.5-2.0 | 2.0-5.0 | 5.0-10 | >10 |
| Fibrinogen (mg/dL) | >150 | 150-125 | 125-100 | 100-75 | <75 |
| BUN (umol/L) | <7.1 | 7.1-14.3 | 14.3-21.4 | 21.4-28.5 | >28.5 |
