# Supplementary material for: Validating the performance of organ dysfunction scores in children with infection: A cohort study
Source: PLoS One. 2024 Jul 19;19(7):e0306172. doi: 10.1371/journal.pone.0306172 (PMC11259267; doi:10.1371/journal.pone.0306172)
Supplement: S2 Table — (DOCX) [file pone.0306172.s012.docx]

**S2 Table. Variables of various scoring models and the number of missing values for each variables.**

| **Variable** | **Model** | | | | | | | | **Number of missing values (N=5356)** |
| --- | --- | --- | --- | --- | --- | --- | --- | --- | --- |
|  | pSOFA | pSOFAal | SIRS | PELOD2 | Sepsis-2 | qSOFA | qSOFAal | PMODS |  |
| Pao2 | yes | yes | no | yes | yes | no | no | yes | 135 |
| Fio2 | yes | yes | no | yes | yes | no | no | yes | 126 |
| Platelet count | yes | yes | no | yes | yes | no | no | no | 116 |
| Bilirubin | yes | yes | no | no | yes | no | no | yes | 162 |
| MAP | yes | no | no | yes | no | yes | no | no | 525 |
| SBP | no | yes | no | no | yes | no | yes | no | 481 |
| Glasgow Coma Score | yes | yes | no | yes | yes | yes | yes | no | 528 |
| Creatinine | yes | yes | no | yes | yes | no | no | no | 221 |
| Heart Rate | no | no | yes | yes | no | yes | yes | no | 207 |
| Respiratory Rate | no | no | yes | yes | no | no | no | no | 471 |
| Leukocyte Count | no | no | yes | yes | no | no | no | no | 115 |
| Temperature | no | no | yes | yes | no | no | no | no | 218 |
| Lactatemia | no | no | no | yes | no | no | no | yes | 106 |
| PaCO2 | no | no | no | yes | yes | no | no | no | 135 |
| Invasive ventilation | no | no | no | yes | yes | no | no | no | 253 |
| Fibrinogen | no | no | no | no | no | no | no | yes | 126 |
| BUN | no | no | no | no | no | no | no | yes | 202 |
