# Supplementary material for: Validating the performance of organ dysfunction scores in children with infection: A cohort study
Source: PLoS One. 2024 Jul 19;19(7):e0306172. doi: 10.1371/journal.pone.0306172 (PMC11259267; doi:10.1371/journal.pone.0306172)
Supplement: S3 Table — (DOCX) [file pone.0306172.s013.docx]

**S3 Table. Performance of pSOFAal vs pSOFA and qSOFAal vs qSOFA**

|  | **pSOFAal** | **pSOFA** | **qSOFAal** | **qSOFA** |
| --- | --- | --- | --- | --- |
| **Scores** |  |  |  |  |
| Survival group[Median(IQR)] | 4.00(3.00, 6.00) | 4.00(3.00, 6.00) | 1.00(1.00, 2.00) | 1.00(1.00, 2.00) |
| Death group[Median(IQR)] | 7.00(5.00, 9.00) | 7.00(5.00, 10.00) | 2.00(1.00, 2.00) | 2.00(1.00, 2.00) |
| **Logistic regression model** |  |  |  |  |
| Without the baseline model[OR(95% CI)] | 1.58(1.51−1.64) | 1.59(1.53−1.65) | 3.74(3.11−4.49) | 4.28(3.65−5.01) |
| With the baseline model[OR(95% CI)] | 1.56(1.50−1.62) | 1.57(1.51−1.63) | 3.58(2.972−4.32) | 4.25(3.60−5.02) |
| **Discrimination** |  |  |  |  |
| Without the baseline model[AUROC(95%CI)] | 0.78(0.75-0.81) | 0.78(0.76-0.81) | 0.66(0.63-0.68) | 0.71(0.68-0.73) |
| With the baseline model[AUROC(95%CI)] | 0.78(0.76-0.81) | 0.79(0.77-0.81) | 0.68(0.66-0.71) | 0.72(0.69-0.75) |
| **Calibration** |  |  |  |  |
| Without the baseline model[Brier(95%CI)] | 6.8(6.4-7.4) | 6.7(6.2-7.2) | 7.8(7.2-8.4) | 7.5(6.9-8.1) |
| With the baseline model[Brier(95%CI)] | 6.8(6.2-7.3) | 6.7(6.1-7.2) | 7.8(7.2-8.3) | 7.4(6.9-8.0) |
