# Supplementary material for: Validating the performance of organ dysfunction scores in children with infection: A cohort study
Source: PLoS One. 2024 Jul 19;19(7):e0306172. doi: 10.1371/journal.pone.0306172 (PMC11259267; doi:10.1371/journal.pone.0306172)
Supplement: S4 Table — (DOCX) [file pone.0306172.s014.docx]

**S4 Table. Subgroup analyses by age group, gender and basic disease for endpoint events of primary outcome in children with 2 or over versus under 2 model points.**

| **Model** | **Total** | **Age** | | **Gender** | | **Basic disease** | |
| --- | --- | --- | --- | --- | --- | --- | --- |
|  |  | **<2y** | **≥2y** | **male** | **female** | **without** | **with** |
| **pSOFA < 2** | 13/507 | 8/331 | 5/176 | 8/298 | 5/209 | 9/400 | 4/107 |
| **pSOFA ≥ 2** | 475/4849 | 260/3168 | 215/1681 | 269/2885 | 206/1964 | 308/3614 | 167/1235 |
| **pSOFAal < 2** | 13/520 | 8/338 | 5/182 | 8/306 | 5/214 | 9/411 | 4/109 |
| **pSOFAal ≥ 2** | 475/4836 | 260/3161 | 215/1675 | 269/2877 | 206/1959 | 308/3603 | 167/1233 |
| **SIRS < 2** | 92/1649 | 84/1517 | 8/132 | 62/1019 | 30/630 | 66/1324 | 26/325 |
| **SIRS ≥ 2** | 396/3707 | 184/1982 | 212/1725 | 215/2164 | 181/1543 | 251/2690 | 145/1017 |
| **PELOD2 <2** | 1/82 | 1/28 | 0/54 | 1/49 | 0/33 | 1/61 | 0/21 |
| **PELOD2 ≥ 2** | 487/5274 | 267/3471 | 220/1803 | 276/3134 | 211/2140 | 316/3953 | 171/1321 |
| **Sepsis-2 < 2** | 66/2104 | 44/1400 | 22/704 | 32/1251 | 34/853 | 46/1617 | 20/487 |
| **Sepsis-2 ≥ 2** | 422/3252 | 224/2099 | 198/1153 | 245/1932 | 177/1320 | 271/2397 | 151/855 |
| **qSOFA < 2** | 152/3469 | 114/2576 | 38/893 | 87/2034 | 65/1435 | 101/2658 | 51/811 |
| **qSOFA ≥ 2** | 336/1887 | 154/923 | 182/964 | 190/1149 | 146/738 | 216/1356 | 120/531 |
| **qSOFAal<2** | 191/3667 | 131/2668 | 60/999 | 108/2156 | 83/1511 | 125/2796 | 66/871 |
| **qSOFAal ≥ 2** | 297/1689 | 137/831 | 160/858 | 169/1027 | 128/662 | 192/1218 | 105/471 |
| **PMODS < 2** | 2/197 | 0/91 | 2/106 | 1/108 | 1/89 | 1/148 | 1/49 |
| **PMODS≥ 2** | 486/5159 | 268/3408 | 218/1751 | 276/3075 | 210/2084 | 316/3866 | 170/1293 |

**Notes:**Events/subgroup were shown for the group aged below 2 years vs. the group aged 2 or over years, the male group vs. the female group, the group with basic disease vs. the group without basic disease, and the total cohort.
