# Supplementary material for: Validating the performance of organ dysfunction scores in children with infection: A cohort study
Source: PLoS One. 2024 Jul 19;19(7):e0306172. doi: 10.1371/journal.pone.0306172 (PMC11259267; doi:10.1371/journal.pone.0306172)
Supplement: Attachment — Submitted filename: Reply letter_plosone.docx [file pone.0306172.s016.docx]

**Reply Letter**

Dear Editors and Reviewers,

On behalf of all the contributing authors, I would like to express our sincere appreciation of your letter and the reviewers’ constructive comments concerning our article titled “Validating the performance of organ dysfunction scores in children with infection: A cohort study” (Manuscript No: PONE-D-23-35521). These comments were all valuable and helpful for improving our article. According to the comments of the associate editor and reviewers, we have made extensive modifications to our manuscript and included additional data to make our results more convincing. In this revised version, changes to our manuscript are highlighted within the document in red text. Point-by-point responses to the comments of the academic editor and two reviewers are listed below.

We would like to thank you for allowing us to resubmit a revised copy of the manuscript, and we greatly appreciate your time and consideration.

Thank you, and best regards.

Yours sincerely,

*Liping Tan*

E-mail: *[tanlp0825@hotmail.com](mailto:tanlp0825@hotmail.com)*

**Point-by-point responses to the comments:**

**Journal requirements:**

1. Please ensure that your manuscript meets PLOS ONE's style requirements, including those for file naming. The PLOS ONE style templates can be found at

https://journals.plos.org/plosone/s/file?id=wjVg/PLOSOne_formatting_sample_main_body.pdf and

<https://journals.plos.org/plosone/s/file?id=ba62/PLOSOne_formatting_sample_title_authors_affiliations.pdf>

Response: We are grateful for the suggestion. According to the style requirements of PLOS ONE, we have made extensive modifications to our manuscript and supplementary material to meet the standards of the journal.

2.Note from Emily Chenette, Editor in Chief of PLOS ONE, and Iain Hrynaszkiewicz, Director of Open Research Solutions at PLOS:

Did you know that depositing data in a repository is associated with up to a 25% citation advantage (https://doi.org/10.1371/journal.pone.0230416)? If you’ve not already done so, consider depositing your raw data in a repository to ensure your work is read, appreciated and cited by the largest possible audience. You’ll also earn an Accessible Data icon on your published paper if you deposit your data in any participating repository (https://plos.org/open-science/open-data/#accessible-data).

Response: We appreciate your valuable feedback. As per your suggestion, we have uploaded the raw data of the manuscript (S1_dataset).

3. Thank you for stating the following financial disclosure:

“The study was supported by Chongqing Science and Technology Bureau and Health Commission Joint Medical Project (2021MSXM025), Chongqing Medical University Graduate Smart Medical Project (YJSZHYX202007), and Program for Youth Innovation in Future Medicine, Chongqing Medical University, Chongqing 400014, China.”

Please state what role the funders took in the study. If the funders had no role, please state: "The funders had no role in study design, data collection and analysis, decision to publish, or preparation of the manuscript."

If this statement is not correct you must amend it as needed.

Please include this amended Role of Funder statement in your cover letter; we will change the online submission form on your behalf.

Response: We appreciate the editor's suggestions. Based on your feedback, we have made the necessary corrections in the corresponding section of the manuscript.

4. Please provide a complete Data Availability Statement in the submission form, ensuring you include all necessary access information or a reason for why you are unable to make your data freely accessible. If your research concerns only data provided within your submission, please write "All data are in the manuscript and/or supporting information files" as your Data Availability Statement.

Response: We sincerely appreciate the valuable comments. According to the editor’s suggestion, we have uploaded the raw data of the manuscript; all the data have been added to the manuscript and/or supporting information files. We also revised our Data Availability Statement as follows: “All the data are in the manuscript and/or supporting information files”.

5. Please include captions for your Supporting Information files at the end of your manuscript, and update any in-text citations to match accordingly. Please see our Supporting Information guidelines for more information: <http://journals.plos.org/plosone/s/supporting-information.>

Response: We are grateful for the editor's advice. Following the specified guidelines, we have made detailed modifications to the supplementary materials, added a list of supporting information at the end of the manuscript, and updated all in-text citations to match accordingly.

**Reviewers' comments:**

**Reviewer #1:** The authors present a retrospective cohort study on the validity of organ dysfunction scores in children with suspected infection admitted to the pediatric intensive care unit. The authors should be commended for their effort to analyse this large study population but I have several concerns.

**Major concerns:**

1. Recently the PODIUM organ dysfunction scoring system has been proposed (PMID: 34970673). I highly recommend the authors assess the possibility to include this scoring system into their analysis to make their analysis more relevant. At the least this new organ dysfunction scoring system should be discussed in their manuscript to keep the discussion of the relevant literature accurate.

Response:

Thank you very much for your thoughtful suggestions. We read this study (PMID: 34970673) in detail and found that PODIUM provides an updated evidence-based foundation for both single- and multiple-organ dysfunction in critically ill children in the PICU. PODIUM includes an organ dysfunction list of 43 criteria for ten systems, including the neurologic, cardiovascular, respiratory, gastrointestinal, acute liver, renal, hematologic, coagulation, endocrine, endothelial and immune systems. Additionally, suggested thresholds for each item and severity grades for some of the items in the PODIUM criteria were provided. Although PODIUM may be complicated and difficult to use as a simple scoring system, it provides a contemporary foundation for researchers to identify and study single- and multiple-organ dysfunction in critically ill children. In our data, the core variable set is also in accordance with the PODIUM list. These new organ dysfunction criteria are noted in the revised manuscript in red font, and the relevant content is as follows (Page 14 Line 7 to 11):

“Recently, the Pediatric Organ Dysfunction Information Update Mandate, which derived an evidence-based organ dysfunction list for ten organs from published literature reviews, provided the latest roadmap for pediatricians to conduct and validate single- or multiple-organ dysfunction in children with sepsis.”

2. The authors state they compare 8 organ dysfunction scores, but alternate SOFA, and alternate qSOFA only swap MAP for SBP. The results of this comparison could be presented as supplementary analysis and IMHO do not qualify for inclusion as separate scores. (the results of the analysis could probably be abbreviated that there was no difference in the use of MAP or SBP).

Response:

We are grateful for the suggestion. To clarify this point, in accordance with the reviewer’s concerns, we revised all the images (in red font) and relevant figures and tables in the manuscript regarding alternate SOFA and alternate qSOFA scores. The results of these comparisons are presented in the supplementary analysis (Table S3). The relevant results have been abbreviated in the manuscript according to the reviewer’s thoughtful suggestion. In the revised manuscript, the alternate SOFA and alternate qSOFA scores were no longer regarded as separate scores for sepsis. The relevant content in the revised manuscript is marked in red font.

3. The authors should also clarify why they included SIRS, which is not an organ dysfunction scoring system and has been removed from the definition of adult sepsis 7 years ago (obviously pediatric definitions are not yet published but will adopt this as well). This is relevant as the authors presumably report on a very sick population (>85% with mechanical ventilation) but only 70% had a SIRS score >=2, which begs the question how relevant this measure could be in their population?

Response:

We are grateful for the suggestion. Since the publication of Sepsis 3.0 in 2016, the SIRS score has no longer been used as an organ dysfunction tool for adults with sepsis because of its poor sensitivity and specificity based on a range of clinical evidence. However, for children, the latest SSC guidelines cannot recommend a specific organ dysfunction tool, which may be partly attributed to insufficient evidence-based data in pediatrics. Although several clinical studies among children revealed that the SIRS score has poor specificity and limited clinical value, this tool has still been employed in the treatment of pediatric organ dysfunction in some areas. This is also the reason why this study included the SIRS score as an assessment tool for organ dysfunction in pediatric patients. Our data also showed that the SIRS score may miss critical patients. Accordingly, we revised the content in the Introduction section. The relevant content is as follows (Page 4, Line 12 to 14):

“Given that the latest SSC guidelines do not recommend a specific organ dysfunction tool for children with sepsis, SIRS was included in this study. ”

4. Discussion: your interpretation of the accuracy, calibration and net benefit of the scores seems a bit euphoric. They can hardly be called excellent and outstanding.

Response:

Thank you for the suggestion. We have made corrections according to the Reviewer’s comments. We have modified these expressions in the discussion, and the changes are shown in red according to the comment. The relevant content is as follows (Page 12, Lines 15 to 17):

“These two scores also revealed good calibration between the prediction and observation and better clinical net benefit prediction than did the other scores for hospital mortality.”

5. Judgement of usefulness of PELOD-2 based on the availability of pupillary dilatation in your database is moot. This can be easily measured and is a failure of the design of the database for the analysis presented here, please expand on the limitations section (e.g. children did not have proven infection, data validity for other settings, etc.).

Response:

Thank you for highlighting this deficiency. Based on your comments, we have expanded the Limitations section, and the revised content is as follows (Page 13, Lines 1 to 3; Page 13, Lines 3 to 5):

“ Between PELOD2 and age-adapted SOFA, the latter is more likely to be applied in clinical practice because it has fewer items.”

“Fourth, the PELOD2 score was calculated without including pupillary dilation because it could not be extracted from the retrospective database. ”

**Minor concerns:**

1. p3, l3. The estimates for sepsis incidence do not correspond with the numbers given in the cited reference.

Response:

Thank you for your careful review. Based on your comments, we have carefully checked the manuscript and corrected the errors accordingly. The revised content is as follows (Page 3 Line 3 to 5):

“It is estimated that 25.2 million children suffer from sepsis annually, and approximately 3.3 million children with sepsis die every year”

2. p3, l5. Reference 3 cited by the authors only concerns adults with sepsis, it has no relevance whatsoever to the statement of the sentence.

Response:

We sincerely appreciate the valuable comments. We have checked the literature carefully and removed Reference 3 accordingly.

3. p3, l11 and p4 l5. the use of the word invalids is inappropriate here (see also above).

Response:

We apologize for the language problems in the original manuscript. As suggested by the reviewer, we have corrected “invalids” to “patients” in red in the manuscript (Page 3 Line 11, Page 4 Line 5).

4. p4, l12. typo, this should probably say SAOD? please check all abbreviations for correctness.

Response:

We apologize for our carelessness. In our resubmitted manuscript, the typo has been revised to “SAOD”. Thank you for your correction.

5. p4, l20. I don't think the word multicentre applies here, please remove. The data are from two sites in the same city and affiliated with the same university.

Response:

Thank you for pointing this out. The term “multicenter” has been corrected to “single center” on page 4, line 21.

6. p4, l20. This is a retrospective study, patients cannot be recruited.

Response:

This was a very large mistake for the overall quality of our article. We apologize for our carelessness. We have corrected this, and we also appreciate your comments. The revised text reads as follows (Page 4 Line 21 to Page 5 Line 2):

“A single-centre retrospective cohort study was performed, and aged four weeks to 18 years who were admitted to the PICU at the Children’s Hospital of Chongqing Medical University (CHCMU) in China with suspected or defined infection from 2015 to 2021 were included.”

7. p6, l1. Supplementary Table 2 does not contain the information the authors refer to.

Response:

We apologize for our carelessness. In our resubmitted manuscript, the error has been corrected. Thank you for your comment.

8. p6, l1-8. This is another example of poor language. The authors didn't develop a score.

Response:

We apologize for the language problems in the original manuscript. We sincerely thank the reviewer for their comments. As suggested by the reviewer, we have changed “developed” to “calculated” (Page 6 Line 5).

9. p6, l1-8. I suggest the authors provide this information as a cross table (i.e. for each measure (e.g. SBP, row) they should indicate whether it was used in the respective score (columns pSOFA-SIRS) or not and add the number of missing values).

Response:

This suggestion is appreciated. As suggested by the reviewer, a cross table was created in the supplementary material (Table S2), in which the row represents each measure, the column represents the respective score, and the separate columns represent the number of missing values.

10. p6, l21. Can the authors please add references to decision curve analysis and clinical impact curve.

Response:

Thank you for the suggestion. We have checked the literature carefully and added references 20and 21 accordingly. The revised references are as follows:

20. Van et.al: Reporting and Interpreting Decision Curve Analysis: A Guide for Investigators. EUR UROL 2018, 74(6):796-804.

21. Kerr et al: Assessing the Clinical Impact of Risk Prediction Models With Decision Curves: Guidance for Correct Interpretation and Appropriate Use. J CLIN ONCOL 2016, 34(21):2534-2540.

11. p6, l22 - p7, l2. Can the authors please clarify what they exactly aimed to do with subgroup analysis, given they had done a multivariate regression model.

Response:

This suggestion is appreciated. As the reviewers noted, in addition to including a multivariate regression model (including age, sex, and underlying diseases) to adjust for confounding factors, we also conducted subgroup analysis of these factors. The purpose of the subgroup analysis was to explore whether the thresholds of various scores (2 points) had obviously different distributions across different subgroups (age, sex, presence or absence of comorbidities). In other words, the latter was a supplementary analysis to the former. There may have been some overlap between the two in terms of statistical inference, making them slightly redundant. Based on the reviewers' suggestions for this manuscript, we made modifications. We simplified the content of the subgroup analysis and moved Figure 4 in the original manuscript to the Supplementary Materials (S4_Fig).

12. p7, l3. Please explain how multiple imputation was done. Also show the rationale for doing so. Most organ dysfunction scores were developed by assuming that missing values indicate that the value was normal (this does obviously does not apply to systematically missing data as the authors had in their database for pupillary dilation).

Response:

This suggestion is appreciated. In this study, data for fewer than 10% of the primary candidate variables were missing; these values were considered missing completely at random, and missing values were processed by multiple imputation (MI). MI was performed by using the *mice* package of R. Patients for whom more than 10% of the predictor variables were missing were considered missing not at random. For pupillary dilation, data that were missing were considered systematic missing data, so these data were not included in the subsequent data analysis.

13. p7, l9. this should be suspected infection.

Response:

We apologize for our carelessness. In the revised manuscript, the error has been corrected.

14. p7, l16. Why is this Suppl. Fig 3? What happened to Suppl. Fig 1 & 2, they are not mentionned.

Response:

We apologize for these incorrect citations in the original manuscript. We have carefully checked the manuscript and corrected the errors accordingly. Fig. S1 was mentioned on page 7, line 7; Fig. S2 was cited on page 7, line 20; and Fig. S3 was mentioned on page 9, line 16.

15. p8, l6-10. This information is given in supplementary figure 3. please add reference and shorten the text accordingly.

Response:

Thank you for the suggestion. As suggested by the reviewer, we added a reference and shortened the text in the corresponding section of the manuscript (Page 9, Lines 14 to 16).

16. p8, l11-15. It would be better to show a graph with mortality by each increase of 1 point of the different scoring systems as a supplementary figure. Then this section can probably be shortened as well.

Response:

We have carefully addressed the reviewer's concerns and plotted a graph with mortality for each increase of 1 point in the different scoring systems, as shown in Figure 4. This graph more clearly visualizes the trend of mortality with increasing score. After discussion, we included this figure in the main text of the revised manuscript and moved Fig. 4 in the previous manuscript to the supplementary material. Based on the main information presented in these two figures, we revised the description of this section in the revised manuscript to make it more concise. The revised parts of the manuscript are as follows (Page 9 Line 18 to 21):

“For the SOFA, pPELOD2, and SEVSEPSIS scores and PMODS, mortality increased with increasing score (Figure 1). For SOFA, PELOD2, PMODS, qSOFA and SEVSEPSIS, patients with 2 or more points had more than a 4-fold increase in mortality than patients with a 2-fold change in SIRS (Fig. S4).”

17. p8, l15-17. The authors presumably report on a very sick population, how is it possible that so many children have a SIRS score <2?

Response:

We are grateful for the suggestion. The SIRS score includes four items: heart rate, respiratory rate, leukocyte count, and temperature. Clinically, in pediatric patients with sepsis shock in the PICU, which is a critical illness, the heart rate may not be abnormal due to resuscitation in the emergency room, the respiratory rate due to mechanical ventilation, the leukocyte count and the temperature. Therefore, the SIRS score may be less than 2. Relevant data analysis in this study also showed this phenomenon, as shown in Figs. 1 and S4. In this cohort, some children in critical condition were receiving mechanical ventilation, but their SIRS score was less than 2. Previous studies have also shown the limited value of the SIRS score in the assessment of acute organ dysfunction in children.

18. p8, l20 - p9, l11. No need to formulate "odds ratio of 1.59 equalling an increase of 59% in the relative odds". This is what the odds ratio tells us already. please remove the unnecessary repetition of information.

Response:

Thank you for your comment. As suggested by the reviewer, we have revised this section in red font in the manuscript, and the context has been revised as follows (Page 10 Line 4 to 9):

“In the cohort, six scores with or without the adjusted baseline model were associated with a similar odds ratio (OR) and 95% confidence interval (95% CI) for in-hospital mortality [SOFA: 1.59(1.53-1.65), 1.57(1.51-1.63); SIRS: 1.46(1.34-1.6), 1.39 (1.26-1.54); PELOD2: 2.1(1.97-2.24), 2.07(1.94-2.21); SEVSEPSIS: 2.71(CI 2.46-2.98), 2.65(2.41-2.92); qSOFA: 4.28(3.65-5.01), 4.25(3.60-5.02); PMODS: 1.44(1.39-1.5), 1.44(1.38-1.49); un- or adjusted model, respectively] (Fig. 2). ”

19. p9, l19-20. This is somewhat surprising. Can you specify what the added value of the organ dysfunction scores was in comparison of mortality prediction with the baseline model (AUC of the base line model).

Response:

We apologize for these incorrect expressions in the original manuscript. The revised text reads as follows (Page 10 Line 22 to Page 11 Line 1):

“For the primary outcome, the AUC of each score adjusted for in the baseline model was similar to that of each score separately (Figure 3).”

20. p9, l22. You mention Brier score here, but it's not discussed in the methods. Please add to methods with proper citation.

Response:

Thank you for your comment. As suggested by the reviewer, we have revised the Methods section in red and added more information. The revised text is as follows (Page 6 Line 22 to Page Line 3):

“Calibration plots were generated to determine the agreement between the predicted and observed outcomes, and Brier scores (BS) were calculated to present the overall performance of all the models(19). ”

21. p10, l3-4. The presentation of Brier scores here does not correspond to the way it's shown in Figure 3.

Response:

Thank you for your comment. We have carefully checked the manuscript and Figure 3 and corrected the errors accordingly. In the original manuscript, the Brier scores in Figure 3 should have been marked “BS*100” so that they could be displayed more clearly.

22. p10, l5. Typo. this should say 5 and not 8.

Response:

We apologize for our carelessness. In the revised manuscript, the error has been corrected.

23. p10, l20-22. Lactate is part of PMODS and PELOD-2, how relevant can subgroup analyses be?

Response:

Thank you for your comment. As an item of the PMODS and PELOD2, lactate should not be employed for subgroup analysis. Therefore, we removed the relevant content for the subgroup analysis using lactate in both the manuscript and Supplemental Figure 4. Given the important value of lactate in critically ill patients and the fact that there is still no clear lactate threshold recommendation in children, this put us at a loss. The comments of the reviewer helped us to clarify, so we constructed a bar plot based on mortality for different lactate levels in this cohort and a scatter plot based on the correlation between lactate and the SOFA score. We found that mortality increased with elevated lactate levels and that there was a positive correlation between lactate levels and SOFA scores. The relevant modifications are as follows (Page 12, Lines 7 to 10):

“The data showed that the mortality of infected children increased with increasing lactate level, and the mortality reached above 30% when the lactate level was above 5 mmol/L (Fig. S5). In addition, lactate levels were positively correlated with SOFA scores (Fig. S6).”

24. p13, l1-5. How did you arrive at a lactate threshhold of 2?

Response:

Thank you for the suggestion. In normal human blood, the concentration of lactate is less than 2 mmol/L. There is still no clear lactate threshold for children with critical illness. As mentioned above, we removed subgroup analysis using a lactate threshold of 2 and instead explored the relationship between lactate levels and mortality.

25. p15, l4-7. if you want to suggest lactate should be used with pSOFA, why didn't you look at this in your data? too many missing values?

Response:

We sincerely appreciate the valuable comments. As suggested by the reviewer, we constructed a scatter plot based on the correlation between lactate and the SOFA score (Fig. S6). We found that there was a positive correlation between the lactate acid concentration and SOFA score. The relevant modifications are as follows (Page 14, Lines 18 to 19; Page 16, Lines 20):

“Our study revealed the lactate levels are an important predictor of hospital mortality in children with infections in the PICU. ”

“A combination of both should be used to identify organ dysfunction earlier in sepsis.”

**Tables:**

Table 1:

1. Please carefully check all number! The percentage for mortality by ethnicity is wrong.

Response:

Thank you for the suggestion. We have carefully checked Table 1, and each number was confirmed. There were 5356 children in the cohort; 4871 were of Han ethnicity (90.9%), and 485 were ethnic minorities (9.1%). There were 4868 children in the survival group, 4422 of whom were of Han ethnicity (90.8%) and 446 of whom were ethnic minorities (9.2%). There were 488 children in the death group, 449 of whom were of Han ethnicity (92%) and 39 of whom were ethnic minorities (8%).

2. I suggest to give units for all lab values in the row names and clearly indicate which measure is shown (n (%) versus median (iqr) or (mean (sd) for normally distributed values, see comment above).

Response:

Thank you for your comment. As suggested by the reviewer, we revised Table 1, presented in red text.

3. please add the number (%) of children receiving inotropes, ecmo or dialysis to help better assess the disease severity in the population (also relevant regarding mortality).

Response:

Thank you for the suggestion. As suggested by the reviewer, we added the number (%) of children receiving vasopressors, ECMO or renal replacement therapy to Table 1 (the final three rows).

**Figures:**

1. Figure 4: Consider showing score values <2 before >= 2, this would be more logical (0, 1, 2 ...)

Response:

Thank you for the suggestion. As mentioned above, we removed subgroup analysis using a lactate threshold of 2. Additionally, we have modified Supplemental Figure 4 to show score values <2 before >= 2.

2. Supplementary Figure 4: Please revise the range shown in the DCA plots. it likely dosen't make sense to show risks of mortality > 20-30%. Also consider grouping DCA and CIC in separate panels, or even separate figures to allow the figures to breath (this is in the supplementar material anyway). Please also don't include the graph interpretation in the figure legend.

Response:

We greatly appreciate the reviewer’s suggestion. We revised the DCA plots according to the reviewer’s comment and grouped DCA and CIC in separate figures (Fig. S7 and S8, respectively). Additionally, graphical interpretations have been provided in the notes.

**General concerns with the manuscript presentation:**

1. The english language is quite poor throughout the mansucript. As PlosOne does not offer copyediting of manuscript. I suggest the authors employ a professional service to correct their manuscript (e.g. the authors use the word invalids, probably to indicate children or sick patients on PICU, however I do not think this word is appropriate in the context of the manuscript). There are many other issues with the language, therefore the recommendation to seek professional support.

Response:

We apologize for the poor language of our manuscript. We employed a professional service, and this manuscript has been edited for proper English language, grammar, punctuation, spelling, and overall style by one or more of the highly qualified native English-speaking editors at AJE. We hope that the revised manuscript is acceptable.

2. please do not repeat all information given in tables or figures in the main text. The authors should reduce such unnecessary repetition (e.g. p7, l10-18; p8, l6-10; or p10, l3-l8).

Response:

We carefully checked this in the manuscript and revised the corresponding sections according to the reviewers' comments to make the sentences more concise and clearer in the manuscript. The relevant modifications are shown in red font.

**Reviewer #2:**

The article is quite interesting, comparing various scores for the prognosis of mortality in the PICU. Strengths include the large sample, which led to a normal or approximately normal distribution of the evaluated scores, and the careful and very informative statistics. Another interesting aspect is that the population is Asian, which fills a gap, as these scores are developed and validated in Western populations.

1. The article needs to be rewritten for the most part, as it presents language problems throughout the text. For example, the use of expressions such as "invalids" to refer to patients often makes the text difficult to understand.

Response:

We apologize for the poor language of our manuscript. We employed a professional service, and this manuscript has been edited for proper English language, grammar, punctuation, spelling, and overall style by one or more of the highly qualified native English-speaking editors at AJE. We hope that the revised manuscript is acceptable.

2. There are also many typographical errors, such as "morality" instead of "mortality" on pg 14, line 6.

Response:

We carefully checked the manuscript and corrected these spelling errors, and the changes are shown in red font. Thank you very much for your suggestions.

3. The excessive use of abbreviations also makes the text difficult to follow, and this needs to be reviewed.

Response:

We sincerely appreciate the valuable comments. We have summarized the abbreviations in the manuscript and spelled out the full words when there were fewer than three occurrences to make the article more readable.
