# Supplementary material for: Validating the performance of organ dysfunction scores in children with infection: A cohort study
Source: PLoS One. 2024 Jul 19;19(7):e0306172. doi: 10.1371/journal.pone.0306172 (PMC11259267; doi:10.1371/journal.pone.0306172)
Supplement: Attachment — Submitted filename: Response to Reviewers.docx [file pone.0306172.s017.docx]

**Reply Letter**

Dear *Jekarl*,

On behalf of all the contributing authors, I would like to express our sincere appreciation for your letter and the reviewers’ constructive comments concerning our article titled “Validating the performance of organ dysfunction scores in children with infection: A cohort study” (Manuscript No: PONE-D-23-35521). The comments were all valuable and helpful for improving our article. According to the comments of the associate editor and reviewers, we have made extensive modifications to our manuscript to make our results more convincing. In this revised version, changes to our manuscript are highlighted in red text. Our point-by-point responses to the comments of the academic editor and two reviewers are listed below. We employed a professional service, and this manuscript has been edited for proper English language, grammar, punctuation, spelling, and overall style by one or more of the highly qualified native English-speaking editors at AJE.

We would like to thank you for allowing us to resubmit a revised copy of the manuscript, and we greatly appreciate your time and consideration.

Thank you, and best regards.

Sincerely,

*Liping Tan*

E-mail: *[tanlp0825@hotmail.com](mailto:tanlp0825@hotmail.com)*

**Point-by-point responses to the comments:**

**Journal requirements:**

Please review your reference list to ensure that it is complete and correct. If you have cited papers that have been retracted, please include the rationale for doing so in the manuscript text, or remove these references and replace them with relevant current references. Any changes to the reference list should be mentioned in the rebuttal letter that accompanies your revised manuscript. If you need to cite a retracted article, indicate the article’s retracted status in the References list and also include a citation and full reference for the retraction notice.

Response: We are grateful for the suggestion. We have reviewed our reference list in the PubMed database and did not find any cited papers that have been retracted from the manuscript.

**Reviewer #3:**

This study is a paper evaluating the performance of the six scoring model using data from a large cohort of pediatric patients. Throughout the paper, there are typing errors and some duplicated sentences. Additionally, there are some points that the authors need to revise.

Despite similar performance between PLEOD2 and SOFA in the conclusion, the reasons why PLEOD2 is not optimal are presented. Please include this information in the abstract (in conclusion section) as well.

Response: We sincerely appreciate your valuable comments. Based on your comments, we carefully checked the manuscript and revised the conclusion section in the abstract accordingly. The revised content is as follows (Page 2, Lines 14 to 17):

“The pSOFA and PELOD2 scores had superior predictive performance for mortality. Given the relative unavailability of items and clinical operability, the pSOFA score should be recommended as an optimal tool for predicting acute organ dysfunction in pediatric sepsis patients. ”

Throughout the abstract and the entirety of the manuscript, there is inconsistency in terminologies. Is "age-adapted SOFA" the same as "SOFA" mentioned in the results section? Is this consistent with "paediatric SOFA" as well? Please ensure uniformity and clarity by using one consistent term.

Response: Thank you for your careful review. In this manuscript, "age-adapted SOFA", "SOFA" and "pediatric SOFA (pSOFA)" have the same meanings. In the revised manuscript, we used the term "pSOFA" consistently throughout. We have thoroughly reviewed the entire manuscript, including the figures, tables and supplementary materials, and have corrected each instance to "pSOFA", shown in red font.

In the abstract, is "severe sepsis" correct, or should it be aligned with the Sepsis-2 criteria or SEVSEPSIS (Table 1) as mentioned in the manuscript? If they differ, please explain the distinction in the introduction. "Sepsis-2" (page 4, line 17) or "sepsis-2" (page 16, line 8)?

Response: We sincerely appreciate the valuable comments. In this manuscript, "severe sepsis", "Sepsis-2" and "SEVSEPSIS" have the same meanings. In the revised manuscript, we used the term "Sepsis-2" consistently throughout. We thoroughly reviewed the entire manuscript, including the figures, tables and supplementary materials, and have corrected each instance to "Sepsis-2", shown in red font.

Additionally, supplementary Table 1 and 2 incorrectly label "PELOOD2." In the "Lactatemia" section of "PELOOD2," please include the units.(mmol/L?)

Response: We apologize for our carelessness. In our resubmitted manuscript, the typo has been revised to “PELOOD2”, and the units (mmol/L) have been added to the "Lactatemia" section of "PELOD2" in Table 1. Thank you for your comment.

Page 3 Ln21 It seems that "age-adapted SOFA" is correct rather than "Adapted SOFA”

Response: Thank you for pointing this out. The term “Adapted SOFA” has been corrected to “age-adapted pSOFA” on page 3, line 22.

Page 5 Ln 19 Systolic Blood Pressure (SBP) should be spelled out before using the abbreviation.

Response: Thank you for the suggestion. In the revised manuscript, the error has been corrected.

Page 7 Lns12- 21 Please exclude any unnecessary parts from the content of the study cohort. It would be advisable to remove the mention of "Han nationality" from both the table and the main text.

Response: Thank you for your comment. As suggested, we have removed the mention of "Han nationality" from both the table and the main text.

Table 1: It was stated in the method that the worst value on the first day of PICU admission was used for scoring. Is this also the case for the CRP and PCT in Table 1? It would be beneficial to accurately describe this.

Response: We sincerely appreciate the valuable comments. For the CRP and PCT levels, the lowest value on the first day of PICU admission was used. In the revised manuscript, the error has been corrected.

Page 9: Lines 11-13 and lines 15-17 contain redundant information. Please review and remove the duplicates.

Response: Thank you for pointing this out. As suggested, we removed duplicate content and revised redundant information in this section (Page 9, lines 8-14).

Page 11 Ln15 It seems that the DCA plot corresponds to Supplementary Figure 7. Please verify and make the necessary correction.

Response: We apologize for our carelessness. In the revised manuscript, the error has been corrected.

Page 11 The content in lines 19-21 on page 11 overlaps with the content in lines 8-11 on page 10. Editing is required to address this duplication.

Response: Thank you for pointing this out. In the revised manuscript, we removed the duplicate content in lines 8-11 on page 10 and preserved the relevant content in lines 19-21 on page 11.

Page 12 In this paper, the association between lactate levels and mortality was analyzed. What the analysis results for inflammatory markers such as PCT or CRP are as presented in Table 1? Furthermore, is there any association between these PCT or CRP markers and six scoring models including SOFA? If there is an association, please describe it.

Response: Thank you very much for your thoughtful suggestions. As suggested, we added the PCT and CRP analysis results, which are presented in Table 1. Additionally, we constructed two scatter plots based on the correlation between PCT or CRP levels and the six scoring models (Fig. S9-10). We found that there was no significant association between them. The relevant description is as follows (Page 11, Lines 20-22, Page 12, Lines 1):

“The levels of the inflammatory markers PCT and CRP were significantly greater in the nonsurviving group than in the surviving group (Table 1). Additionally, there was a relatively weak association between PCT or CRP levels and the six scoring systems (adjusted R2 ranging from 0.02 to 0.17) (S9 Fig, S10 Fig). ”

Page 14 Lns 2-5: Please add a reference for lines 2-5 on page 14.

Response: Thank you for the suggestion. As suggested, we added a reference to the corresponding section of the manuscript (Page 13, Line 20).

Page 15 Lns 4-7: Please add a reference for lines 4-7 on page 15.

Response: Thank you for the suggestion. As suggested, we added two references to the corresponding section of the manuscript (Page 14, Line 22).

Page 15 Ln13: The sentence at line 13 on page 15 is unnecessary. Please delete it.

Response: Thank you for pointing this out. In the revised manuscript, we removed the unnecessary text: “Subgroup analyses by age, sex and comorbidities were also performed”.
